# Pathogenic TDP‐43 accelerates the generation of toxic exon1 HTT in Huntington's disease knock‐in mice

**DOI:** 10.1111/acel.14325

**Published:** 2024-08-26

**Authors:** Dazhang Bai, Fuyu Deng, Qingqing Jia, Kaili Ou, Xiang Wang, Junqi Hou, Longhong Zhu, Mingwei Guo, Su Yang, Guohui Jiang, Shihua Li, Xiao‐Jiang Li, Peng Yin

**Affiliations:** ^1^ State Key Laboratory of Bioactive Molecules and Druggability Assessment, Guangdong Key Laboratory of non‐human Primate Research, Guangdong‐Hongkong‐Macau Institute of CNS Regeneration Jinan University Guangzhou Guangdong China; ^2^ Department of Neurology, Affiliated Hospital of North Sichuan Medical College Institute of Neurological Diseases, North Sichuan Medical College Nanchong Sichuan China; ^3^ Shenzhen Institute for Drug Control, Shenzhen Testing Center of Medical Devices In Vitro Diagnostic Reagents Testing Department Shenzhen Guangdong China

**Keywords:** aberrant splicing, Huntington's disease, mislocalization, TDP‐43

## Abstract

Huntington's disease (HD) is caused by a CAG repeat expansion in exon1 of the *HTT* gene that encodes a polyglutamine tract in huntingtin protein. The formation of HTT exon1 fragments with an expanded polyglutamine repeat has been implicated as a key step in the pathogenesis of HD. It was reported that the CAG repeat length‐dependent aberrant splicing of exon1 *HTT* results in a short polyadenylated mRNA that is translated into an exon1 HTT protein. Under normal conditions, TDP‐43 is predominantly found in the nucleus, where it regulates gene expression. However, in various pathological conditions, TDP‐43 is mislocalized in the cytoplasm. By investigating HD knock‐in mice, we explore whether the pathogenic TDP‐43 in the cytoplasm contributes to HD pathogenesis, through expressing the cytoplasmic TDP‐43 without nuclear localization signal. We found that the cytoplasmic TDP‐43 is increased in the HD mouse brain and that its mislocalization could deteriorate the motor and gait behavior. Importantly, the cytoplasmic TDP‐43, via its binding to the intron1 sequence (GU/UG)n of the mouse *Htt* pre‐mRNA, promotes the transport of exon1‐intron1 *Htt* onto ribosome, resulting in the aberrant generation of exon1 Htt. Our findings suggest that cytoplasmic TDP‐43 contributes to HD pathogenesis via its binding to and transport of nuclear un‐spliced mRNA to the ribosome for the generation of a toxic protein product.

## Graphical abstract

The cytoplasmic TDP‐43 is increased in HD KI mouse brain, and this mislocalization could deteriorate the motor and gait behavior in HD KI mice. The reason is, the cytoplasmic TDP‐43, binding to the unique sequence (GU/UG)n of the mouse *Htt* pre‐mRNA, promotes the abnormal translation of the un‐spliced exon1‐intron1 Htt, resulting in the generation of exon1 Htt toxic protein.

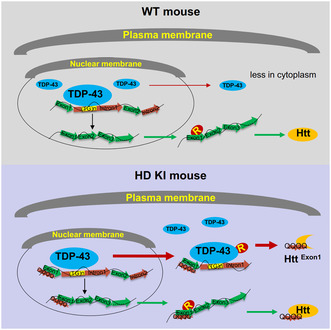

**Abbreviations**
AAV: Adeno‐associated virus
ALS: amyotrophic lateral sclerosis
EX1: exon 1
EX2: exon 2
FISH: fluorescent in situ hybridization
FTD: frontopemporal dementia
HD: Huntington's disease
HTT: huntingtin
In1: intron 1
KI: knock‐in
NLS: nuclear localization signal
polyQ: polyglutamine
RIP: RNA immunoprecipitation
RRMs: RNA‐recognition motifs
RT‐PCR: Reverse transcription PCR
TDP‐43: trans‐active response DNA‐binding protein of 43
UBC: ubiquitin C
WT: wild type

## 1 INTRODUCTION

Huntington's disease (HD) is a hereditary neurodegenerative disorder that manifests with movement disturbances, psychiatric changes, and cognitive decline (Bates et al., 2015). It is caused by an unstable CAG repeat expansion in exon1 of the huntingtin gene (*HTT*), which is translated to an abnormally long polyglutamine (polyQ) stretch in the HTT protein (MacDonald et al., 1993). The expanded polyQ repeats lead to aggregation of mutant HTT and the selective neuronal cell loss in the striatum, cortex and other brain regions in HD patients (Vonsattel et al., 1985; Waldvogel et al., 2015). The *HTT* gene contains 67 exons that encode a 350‐kDa protein (MacDonald et al., 1993), and three full‐length *HTT* mRNA isoforms have been found to be produced by alternative polyadenylation in the 3′untranslated region (3′UTR) (Romo et al., 2017). Also, in the context of an expanded CAG repeat, two small transcripts that contain exon1 and intron1 sequences (*HTT*1a) are produced by the incomplete splicing of the *HTT* transcript (Neueder et al., 2017; Sathasivam et al., 2013). Generation of these aberrant transcripts occurs when one of two cryptic polyadenylation (polyA) signals in intron1 becomes activated. There are cryptic polyA signals located at 2710 and 7327 bp sites in intron1 of human *HTT* (Neueder et al., 2017; Sathasivam et al., 2013) and 680 and 1145 bp in intron1 of mouse *Htt* (Sathasivam et al., 2013). In HD knock‐in (KI) mice, the pathological CAG repeat is integrated into the mouse *Htt* gene to express full‐length mutant Htt at the endogenous level. Thus, the HD KI mice carry the mutation in its appropriate genetic and protein context, making them the most faithful genetic model of HD (Lin et al., 2001; Menalled et al., 2003; Menalled et al., 2012). Also, HD KI mice exhibit more slow progression and development of behavioral, pathological, cellular, and molecular abnormalities (Heng et al., 2007; Menalled, 2005; Menalled et al., 2009). In HD KI mice, *Htt* is subjected to alternative mRNA processing in a CAG repeat length‐dependent manner, in which, more aberrant transcripts are generated when the longer repeats are present (Sathasivam et al., 2013). Importantly, the aberrant *Htt*1a mRNA is translated to produce the highly pathogenic and aggregation‐prone exon1 Htt protein (Mangiarini et al., 1996; Scherzinger et al., 1997). It was recently indicated that the human *HTT* mRNA in YAC128 mice also undergoes alternative processing to produce *HTT*1a, and that the small transcript was exported to the cytoplasm and translated to produce the exon1 HTT protein (Fienko et al., 2022).

Pathogenic expansion of CAG repeats has also been found in frontotemporal dementia (FTD) and amyotrophic lateral sclerosis (ALS) (Dewan et al., 2021; Tazelaar et al., 2020). Dysregulation of the trans‐active response DNA‐binding protein of 43 kDa (TDP‐43) is responsible of approximately 97% of all ALS cases (Prasad et al., 2019). As a nucleo‐cytoplasmic shuttling and DNA/RNA binding protein, TDP‐43 is predominantly and normally localized in the nucleus where it regulates RNA transcription (Swain et al., 2016) and splicing (Fiesel et al., 2012; Tollervey et al., 2011). When mislocalized to the cytoplasm in disease progressions, TDP‐43 associates with a plethora of RNA‐containing complexes including stress and transport granules (Liu‐Yesucevitz et al., 2010; Salajegheh et al., 2009; Vogler et al., 2018) as well as protein complexes devoid of RNA (Gasset‐Rosa et al., 2019; Mann et al., 2019), dysregulates stress granule dynamics (Dewey et al., 2012; Fernandes et al., 2018; Khalfallah et al., 2018; McDonald et al., 2011), and affects the axonal or dendritic mRNA's localization and translation (Alami et al., 2014; Chu et al., 2019; Coyne et al., 2014). Correspondingly, TDP‐43 has also been shown to influence the translation of specific mRNAs, as a negative or positive regulator (Chu et al., 2019; Coyne et al., 2014; Coyne et al., 2017; Majumder et al., 2012; Neelagandan et al., 2019), which contributes to neurodegeneration (Bjork et al., 2022; Neelagandan et al., 2019). Mislocalization of TDP‐43 from the nucleus to the cytoplasm, a pathological hallmark of FTD and ALS, was also detected in HD patients and mouse models (Sanchez et al., 2021; Tada et al., 2012). Given that mutant HTT can be produced by aberrant RNA splicing, it would be interesting to investigate whether the pathogenic TDP‐43 affects *HTT* mRNA transport and expression, leading to the production of toxic HTT products. In the current study, we found that cytoplasmic TDP‐43 is increased in HD KI mice and could deteriorate the motor and gait behavior in HD KI mice. The cytoplasmic TDP‐43 binds to the unique sequence (GU/UG)n in the CAG expanded *Htt* exon1‐intron1 mRNA, facilitating its transport to the ribosome and generation of mutant exon1 Htt in HD KI mice. Our findings suggest that cytoplasmic TDP‐43 contributes to HD pathogenesis by binding the un‐spliced transcripts and transporting them to the ribosomes to produce exon1 mutant Htt.

## 2 RESULTS

### 2.1 The cytoplasmic mislocalization of TDP‐43 promoted the generation of mutant exon1 Htt

**FIGURE 1:**
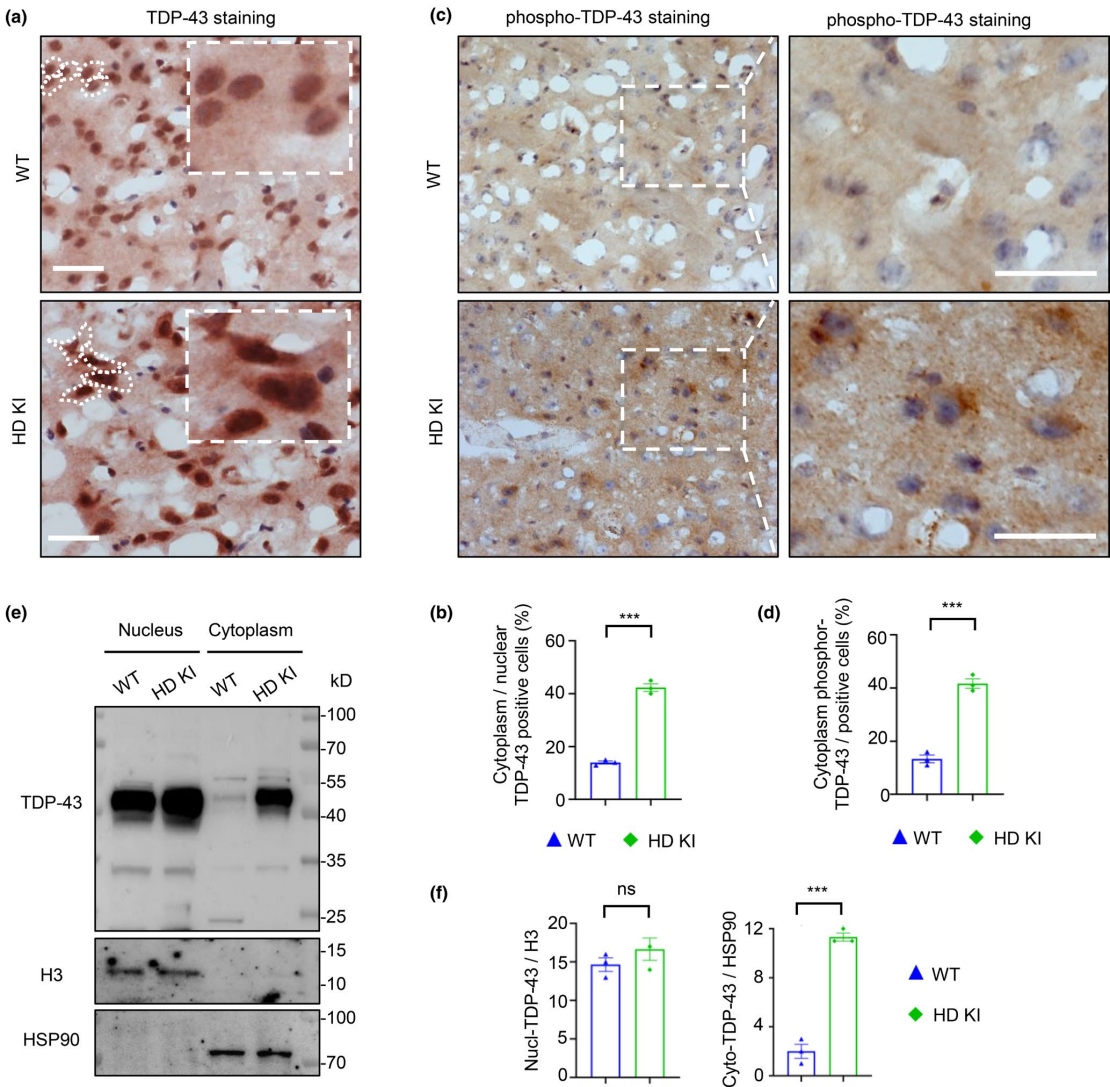
Increased cytoplasmic TDP‐43 in HD KI mice. (a) Immunohistochemical staining with anti‐C‐terminal‐TDP‐43 showed that endogenous TDP‐43 located in the nucleus in the wild‐type (WT) mouse striatum, and the cytoplasmic TDP‐43 is increased in the HD KI mouse striatum. Scale bar: 40 μm. (b) Quantitative analysis of the ratio of cytoplasmic TDP‐43 to nuclear TDP‐43. A total of 20 random fields (63×) in each section were examined, and the data (mean ± SEM) were obtained from three independent experiments (*n* = 3 for each group, 6 months old, ***p < 0.001). (c) Immunohistochemical staining with anti‐phospho‐TDP‐43 (Ser409/Ser410) showed that the cytoplasmic mislocalization of TDP‐43 is in the HD KI mouse striatum, as compared with the WT mouse. Scale bar: 40 μm. (d) Quantitative analysis of the ratio of cytoplasm phospho‐TDP‐43 to the total nucleus positive cells. A total of 20 random fields (63×) in each section were examined, and the data (mean ± SEM) were obtained from three independent experiments (*n* = 3 for each group, 6 months old, ***p < 0.001). (e) Western blotting analysis showing an increase of cytoplasmic TDP‐43 in the HD KI mouse striatum as compared with wild‐type (WT) mouse. HSP90 and Histone H3 are cytoplasmic and nuclear marker proteins, respectively. (f) The relative levels of TDP‐43 in the cytosolic (Cyto) (ratios of cytoplasmic TDP‐43 to HSP90) and the nuclear (ratios of nuclear TDP‐43 to Histone H3) fractions. The data are mean ± SEM (*n* = 3 animals per group, 6 months old; ***p < 0.001; ns, non‐significant).

Although TDP‐43 was found in the cytoplasmic stress granules in R6/2 mice that overexpress transgenic exon1 mutant Htt (Sanchez et al., 2021), it remains unknown whether cytoplasmic TDP‐43 is increased in HD KI mice that express full‐length mutant Htt at the endogenous level and whether cytoplasmic TDP‐43 can exacerbate the phenotypes of HD mice. To address these issues, we performed the immunohistochemical staining (Figure 1a,b), using a polyclonal antibody against the C‐terminal amino acids of TDP‐43, which could recognize the cytoplasmic mislocalized TDP‐43 (Yin et al., 2019, 2021), or a monoclonal antibody against the phosphorylation of C‐terminal TDP‐43 in residues 409/410 (Figure 1c,d), moreover, the western blotting for the cytoplasmic and nuclear fractions of the wild‐type (WT) and HD KI mice at 6 months old (Figure 1e,f). All the results revealed that the endogenous TDP‐43 was mostly located in the nucleus in the WT mouse striatum, but mislocalized in the cytoplasm of age‐matched HD KI mouse brain.

**FIGURE 2:**
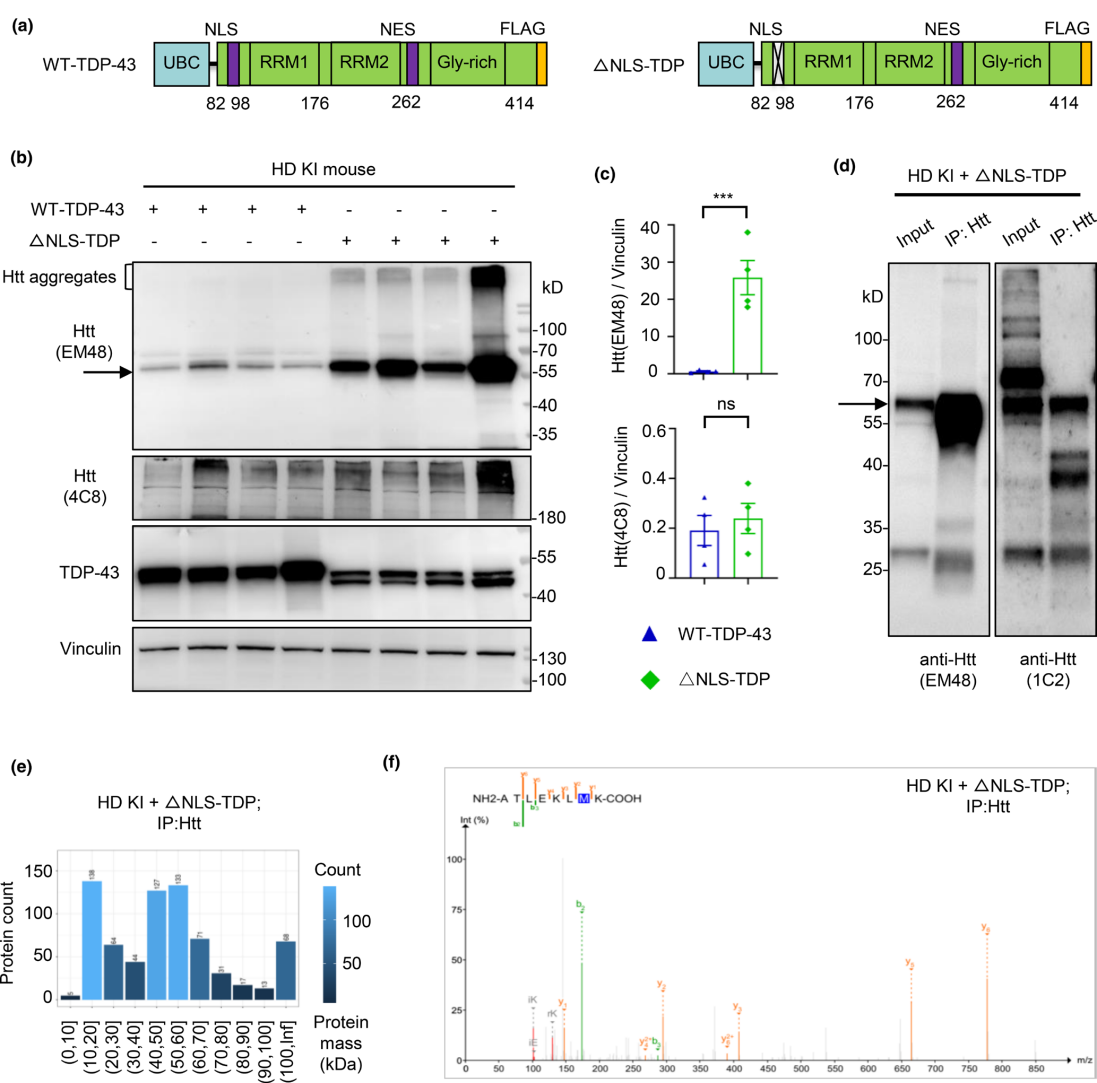
Cytoplasmic TDP‐43 increases the generation of exon1 Htt in HD KI mice. (a) The plasmid DNA structures for expressing full‐length TDP‐43 and the cytoplasmic TDP‐43 with deletion of nuclear localization signal (NLS) (ΔNLS‐TDP‐43), under the same UBC promoter based on the pRK or AAV vector. (b) Western blotting analysis shows that the expression of AAV‐ΔNLS‐TDP‐43, but not AAV‐WT‐TDP‐43, in 3.5 months old HD KI mouse striatum for 1 month could generate more exon1 Htt (between 55 and 70 kDa, the arrow pointed). Note that ΔNLS‐TDP‐43 is slightly smaller than the full‐length TDP‐43 because of the deletion of nuclear localization signal (*n* = 4 for each group). The large band (>100 kDa) may represent the aggregated proteins. (c) Quantitative analysis of the ratios of the generated exon1 Htt or full‐length Htt bands (arrow) to vinculin. The data (mean ± SEM) were obtained from three independent experiments (*n* = 4 for each group; ***p < 0.001; ns, non‐significant). (d) Western blotting of the immunoprecipitated exon1 Htt fragments (arrow), by mEM48 antibody, in 3.5 months old HD KI mice striatum, with injection of AAV‐ΔNLS‐TDP‐43 for 1 month, showing that exon1 Htt was probed by the EM48 or 1C2 antibodies. Note that the expression of exon1 Htt was abundant in HD KI mouse. (e) Mass spectrometry of the immunoprecipitated exon1 Htt revealed the enrichment signals in both 40 to 50 kDa and 50 to 60 kDa peptides in HD KI mice, injected with AAV‐ΔNLS‐TDP‐43. (f) The sequences of the precipitated Htt were verified to contain N‐terminal Htt amino acids ATLEKLMK in HD KI mice.

Next, to explore whether the pathogenic TDP‐43 in the cytoplasm plays a causative role in HD pathogenesis, we expressed the full‐length TDP‐43 (WT‐TDP‐43) and the cytoplasmic TDP‐43 (ΔNLS‐TDP‐43) without nuclear localization signal in Neuro‐2a cell line (Figure 2a), which was also described in our early study (Yin et al., 2021). The immunofluorescence staining indicates that the exogenous WT full‐length TDP‐43 was localized in the nucleus, whereas ΔNLS‐TDP‐43 was distributed in the cytoplasm (Figure S1). Then we injected AAV‐WT‐TDP‐43 or AAV‐ΔNLS‐TDP‐43 into the striatum of HD KI mice at 3.5 months of age. One month after injection, western blotting analysis of the injected brain tissues demonstrated that the cytoplasmic TDP‐43 expression increased the level of N‐terminal Htt at 55 kDa and 70 kDa (Figure 2b,c), which is equivalent to mutant exon1 Htt in HD KI mouse brain and can be recognized by Htt EM48 and polyQ 1C2 antibodies (Figure 2d) (Yang et al., 2020). Although the truncated mutant Htt‐exon1 band was also illustrated in many previous reports (Mangiarini et al., 1996; Neueder et al., 2017; Sathasivam et al., 2013; Scherzinger et al., 1997), it remains to be verified that this band represents exon1 Htt. We precipitated the endogenous Htt using the mEM48 antibody (Figure 2d), and analyzed the immunoprecipitated proteins at 55‐70 kDa position via mass spectrometry. The protein database search revealed the enrichment signals between 40 kDa and 60 kDa groups (Figure 2e) and the presence of the Htt amino acids (ATLEKLMK), which follow the translational initiation site Met in Htt exon1 (Figure 2f and Figure S2).

### 2.2 Cytoplasmic TDP‐43 could deteriorate the motor behavior and gait patterns in HD KI mice

**FIGURE 3:**
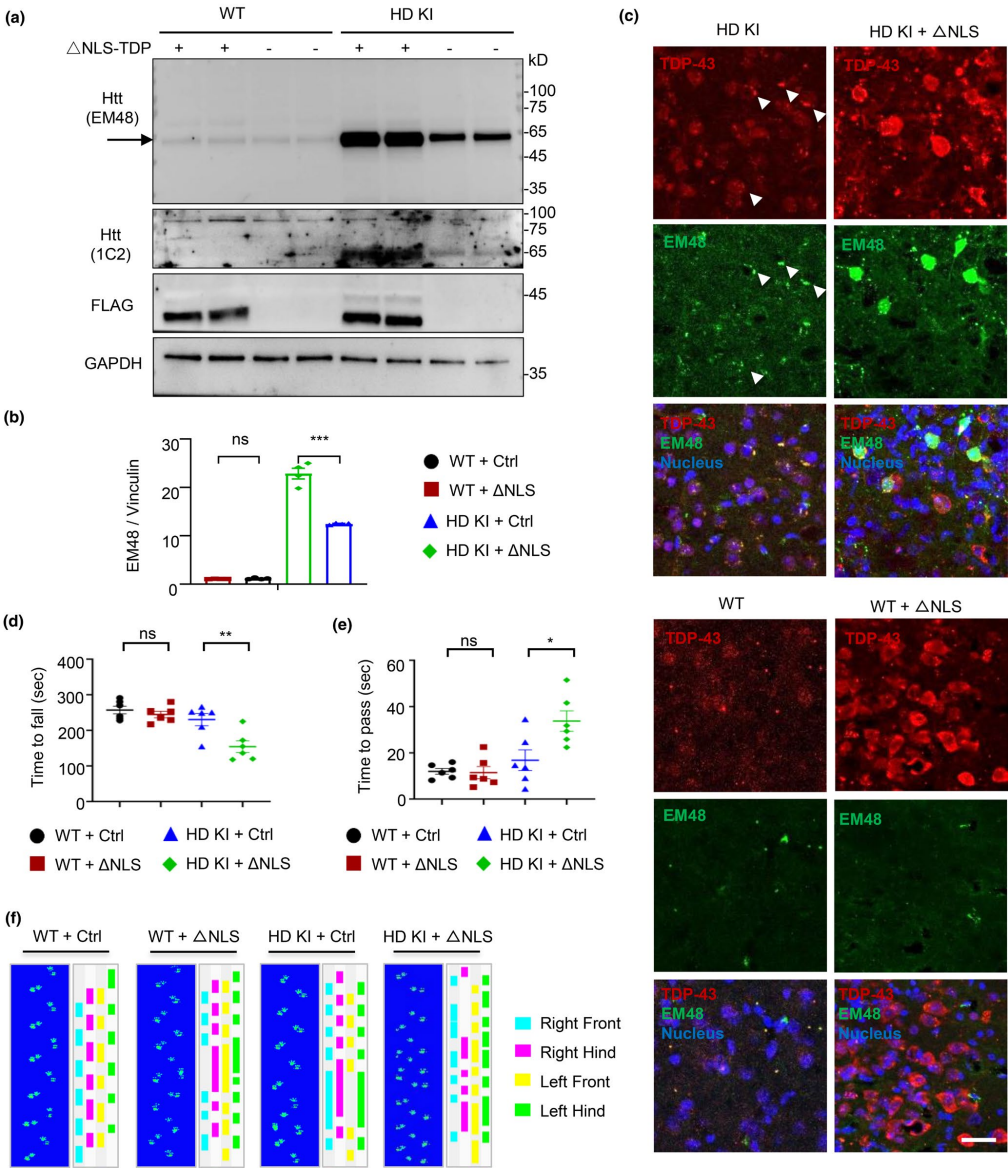
Cytoplasmic TDP‐43 exacerbates the motor function deficit in HD KI mice. (a) Western blotting analysis shows that the expression of AAV‐ΔNLS‐TDP‐43 in HD KI mice for 1 month increased exon1 mutant Htt (about 65 kDa) that was detected by mouse mEM48 or 1C2 antibodies. The 3.5 months old HD KI mice were used for AAV virus injection. Note the similar levels of ΔNLS‐TDP‐43 was detected by anti‐FLAG in the striatum of WT and HD KI mouse. (b) Quantitative analysis of the ratios of exon1 Htt to vinculin on the western blots. The data (mean ± SEM) were obtained from three western blotting experiments (*n* = 4 animals per group; ***p < 0.001). (c) Immunofluorescent double‐staining with anti‐TDP‐43 and anti‐Htt (mEM48) showing that some endogenous TDP‐43 (red) co‐exists with Htt aggregates (green) in the cytoplasm in the striatum of HD KI mice at 3.5 months of age (arrows). The exogenous ΔNLS‐TDP‐43 (red) is distributed in the cytoplasm in the HD KI and WT mice. Scale bar: 40 μm. (d) Rotarod performance displayed the reduced motor function in HD KI, but not WT mice injected with AAV‐ΔNLS‐TDP‐43 in the striatum for 1 months. AAV‐vector injection served as a control (*n* = 6 mice per group; **p < 0.01; ns, non‐significant). (e) The balance beam test of HD KI, but not WT mice, injected with AAV‐ΔNLS‐TDP‐43 showing reduced motor function (*n* = 6 mice per group for the control and ΔNLS‐TDP injection; *p < 0.05; ns, non‐significant). (f) The gait apparatus is outlined and a representative image is shown. Dynamic parameters for gait analysis in ΔNLS‐TDP‐43 expressed in HD KI or WT mouse. Section from original footprints recorded from the animal running, after AAV‐ΔNLS‐TDP‐43 or AAV control injection.

Since the HD KI mouse brain shows the increased level of cytoplasmic TDP‐43, we wonder if cytoplasmic TDP‐43 contributes to HD pathogenesis. To this end, we performed the brain injection of AAV‐ΔNLS‐TDP‐43 (cytoplasmic TDP‐43) into the striatum of HD KI mice at 3.5 months of age. Western blotting analysis of multiple samples showed a marked increase of exon1 mutant Htt in the HD KI mouse striatum that had been injected with the cytoplasmic TDP‐43 for one month (Figure 3a,b and Figure S3a), but not by the injection of AAV‐WT‐TDP‐43 (Figure S3b). Furthermore, we found that the injection of the AAV‐ΔNLS‐TDP‐43 into WT mouse did not generate an obvious exon1 mutant Htt band as compared with HD KI mouse (Figure 3a,b). The immunofluorescent double‐staining with anti‐TDP‐43 and anti‐Htt (mEM48) of the WT, HD KI and the ΔNLS‐TDP‐43 injected individuals, revealed that the endogenous TDP‐43 was mostly located in the nucleus of WT mouse striatum, but mislocalized in cytoplasm of age‐matched HD KI mouse, and co‐existed with Htt aggregates especially (Figure 3c). In addition, the exon1 mutant Htt accumulation was promoted by cytoplasmic TDP‐43 in HD KI mouse striatum, but not in WT mouse (Figure 3c). The AAV‐ΔNLS‐TDP‐43 was also injected into prefrontal cortex to compare the selective contribution of cytoplasmic TDP‐43 on exon 1 Htt. The result showed that cytoplasmic TDP‐43 increased the generation of exon 1 Htt in both of the prefrontal cortex and striatum of HD KI mice as compared with other proteins, but more exon1 Htt production and its aggregate were seen in the striatum (Figure S4). We also found that AAV‐ΔNLS‐TDP‐43 did not significantly affect the age‐related hallmarks (p21^Cip1/Waf1^ and Sirt1) and autophagy related proteins (LC3 or Beclin1) (Figures S4 and S5).

HD KI mice develop age‐dependent phenotypes with deficits in open field, climbing, sensorimotor activity, wheel running, motor learning, and anxiety, which become more obvious after 6 months (Hickey et al., 2008; Menalled et al., 2003). Expressing cytoplasmic TDP‐43 could exacerbate these age‐related defects in rotarod performance (Figure 3d) and balance beam test (Figure 3e) despite no influence on body weight as compared with WT mice (Figure S6a). The gait performance assay, which was assessed by analyzing the footprint pattern of mice while walking along a narrow corridor of catwalk equipment (Xu et al., 2019), revealed that HD KI mice expressing cytoplasmic TDP‐43 lacked a normal, uniform, alternating left–right step pattern as compared with the control HD KI mice receiving the control AAV injection or WT mice injected with AAV‐ΔNLS‐TDP‐43 (Figure 3f). Three‐dimensional graphs of the gait pattern boxed by the dots also support that cytoplasmic TDP‐43 deteriorated the motor function of HD KI mice (Figure S6b).

**FIGURE 4:**
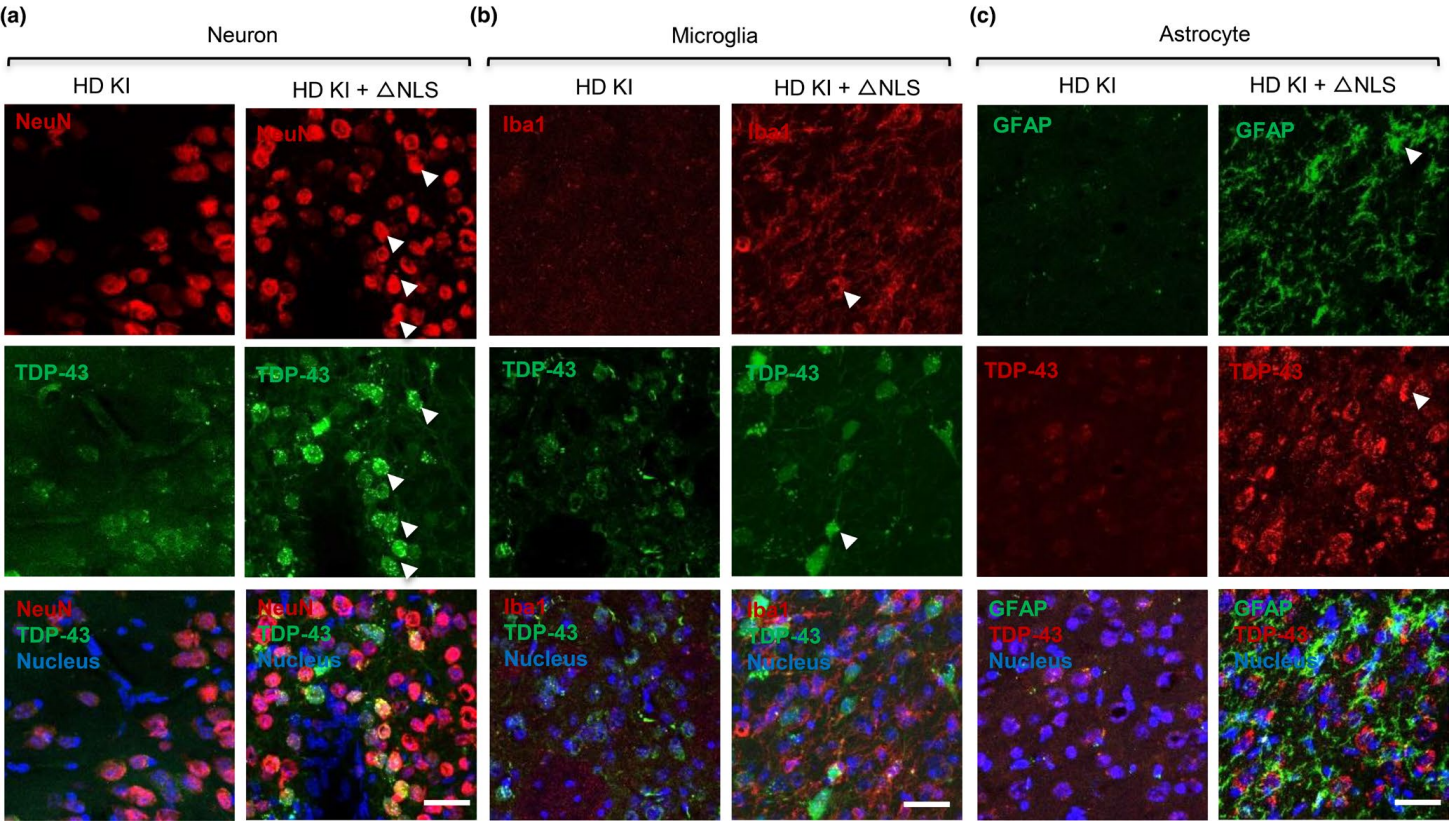
The expression of cytoplasmic TDP‐43 in HD KI mice. (a) Immunofluorescent double‐staining with anti‐TDP‐43 (green) and anti‐NeuN (red) showing that cytoplasmic TDP‐43 is abundantly expressed in neuronal cells of the HD KI mouse brain. No obvious neuronal loss was seen in AAV‐ΔNLS‐TDP‐43 infected HD KI mouse as compared with HD KI mice without AAV‐ΔNLS‐TDP‐43. (b, c) Immunofluorescent double‐staining with anti‐TDP‐43 and anti‐Iba1 (red in b), or anti‐GFAP (green in c), showing that the active glial cells (microglia in b, and astrocyte in c) were promoted by the AAV‐ΔNLS‐TDP‐43 in the striatum of HD KI mouse. Scale bar: 40 μm.

HD is also characterized by the age‐dependent accumulation of misfolded proteins and neurodegeneration. The production of exon1 mutant Htt is identified as a crucial step in the aggregation of Htt and the development of HD‐related pathology (Bates et al., 2015; Machiela & Southwell, 2020; Tallaksen‐Greene et al., 2014; Yang et al., 2020). Our study revealed that the introduction of cytoplasmic TDP‐43 in HD mice can stimulate the production of exon1 mutant Htt and promotes age‐related motor impairments in HD. Furthermore, double immunoflurescent staining showed that cytoplasmic TDP‐43 is more abundantly expressed in neuronal cells than glial cells in the HD KI mouse brain (Figure 4). This is also consistent with the previous finding that TDP‐43 is more stably expressed in neuronal cells than glial cells when it is expressed under the UBC promoter in the mouse brain (Yan et al., 2014). However, we did not see that ΔNLS‐TDP‐43 could significantly increase neuronal loss in the HD mouse striatum despite increased reactive glial cells (Figure 4). This may be related to the fact that HD KI mice do not show overt neuronal degeneration (Crook & Housman, 2011; Levine et al., 2004).

### 2.3 Cytoplasmic TDP‐43 binds un‐spliced *Htt* exon1‐intron1 mRNA

**FIGURE 5:**
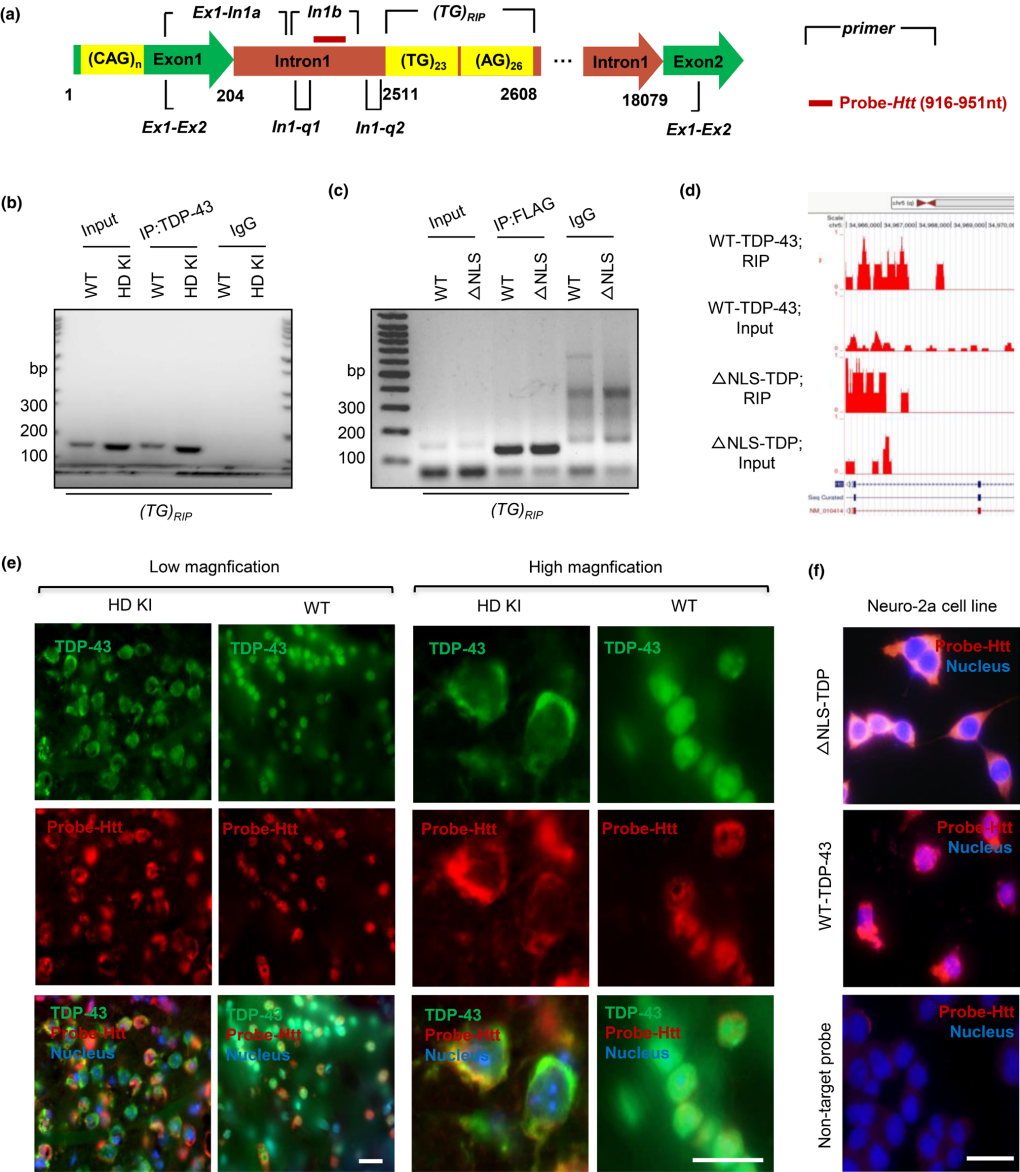
Cytoplasmic TDP‐43 binds un‐spliced *Htt*‐exon1 intron RNA and brings it to the cytoplasm. (a) A schematic diagram for amplifying different *Htt* exon1‐intron regions including exon1, intron1 and (TG)n motif using primers specific to mouse *Htt* sequences. Note that a putative RNA binding conserved sequence (GU/UG)_23_ motif (2511 nt to 2608 nt) was identified in the intron1 region, and a (AG/GA)_26_ tail was indicated at the downstream of (GU/UG)_23_. The specific probe of Probe‐*Htt* (916 nt‐951 nt) labeling Cy3 used for in situ hybridization (FISH) was localized close to the (GU/UG)_23_ motif, which was red lined. (b) The mouse *Htt* (TG)_RIP_ primer was used to detect the enriched (GU/UG)_23_ segment in TDP‐43 immunoprecipitates from the HD KI or WT mouse striatum. IgG served as control for the immunoprecipitation and RT‐PCR. More PCR products with (GU/UG)_23_ motif were observed in HD KI than in WT mouse. (c) The mouse *Htt* (TG)_RIP_ primer was used to detect the enriched (GU/UG)_23_ segment in ΔNLS‐TDP‐43 or WT‐TDP‐43 transfected Neuro‐2a cell line. Although the highly repeated (TG)_23_ affected the amplification efficiency in the input, (GU/UG)_23_ segments were enriched in TDP‐43 immunoprecipitates from the both TDP‐43 transfected cells. The IgG group was the negative control for the RT‐PCR process. (d) The UCSC genome browser was used to analyze the *Htt* transcript reads of TDP‐43 immunoprecipitates. The (GU/UG)_23_ segment on the mouse *Htt* gene (NM_010414), localized on chromosome 5: (34919084 … 35069878), was successfully captured and amplified by deep sequencing. (e) Micrograph images of the FISH experiment with double staining of Cy3‐labeled RNA probes (red) targeting *Htt* intron1 pre‐mRNA, and C‐terminal TDP‐43 antibody. Nuclei were stained with DAPI (blue) dye. Note that endogenous *Htt* pre‐mRNAs, labeled by Probe‐*Htt* that binds the region containing the (GU/UG)_23_ motif, are co‐localized in the cytoplasm with TDP‐43 in HD KI mouse. Scale bars: 40 μm. (f) Micrograph images of the Cy3‐labeled RNA probes (red) targeting *Htt* intron1 pre‐mRNA in Neuro‐2a cell line. Nuclei were stained with DAPI (blue) dye. Note that endogenous *Htt* pre‐mRNAs, labeled by Probe‐Cy3 that binds the region containing the (GU/UG)_23_ motif, are localized in the cytoplasm in cells expressing ΔNLS‐TDP‐43, but in the nucleus in cells expressing WT‐TDP‐43. The control was staining with a Cy3‐labeled non‐target probe. Scale bars: 40 μm.

Exon1 mutant Htt has been found to be pathogenic in various HD mouse models (Yang et al., 2020). Since aberrant spliced *HTT* exon1‐intron1 mRNA, such as *HTT*1a, is present in HD KI mice and can be translated to exon1 mutant Htt in HD mice (Sathasivam et al., 2013) and in post‐mortem brains and fibroblast cultures from HD patients (Ly et al., 2022; Neueder et al., 2017), we wanted to explore whether cytoplasmic TDP‐43 can promote the generation of *Htt* exon1‐intron1 mRNA. We identified a putative TDP‐43 binding sequence (GU/UG)_23_ motif in 2511 to 2608 nt of mouse *Htt* intron1 with a (AG/GA)_26_ tail downstream of (GU/UG)_23_ (Figure 5a). As previously reported, the interaction of TDP‐43 with the UG/TG repeats regulates the expression of the target genes (Buratti & Baralle, 2001; Francois‐Moutal et al., 2019; Kuo et al., 2014; Polymenidou et al., 2011), and the (UG)n repeats having more than 6 units would likely to interact with TDP‐43 (Chang et al., 2012; Mompean et al., 2016; Qin et al., 2014). Therefore, we used the TDP‐43 antibody for immunoprecipitation of TDP‐43 from the mouse striatum (Figure S7) and performed RT‐PCR to detect RNAs associated with TDP‐43. We designed the primers to detect TDP‐43‐associated mRNAs including the (GU/UG)_23_ motif (Table S1). This RNA immunoprecipitation (RIP) assay showed the interaction of aberrant intron1 transcript consisting of 2511–2606 nt and GU/UG motif (TG)_RIP_ with precipitated TDP‐43 from HD KI mice as compared with WT mice (Figure 5b). Overexpression of WT‐TDP‐43 and ΔNLS‐TDP‐43 in Neuro‐2a cells resulted in a similar level of (TG)_RIP_ in the precipitated TDP‐43 revealed by RT‐PCR and sequencing, indicating that TDP‐43 binds to this aberrant transcript regardless of its nuclear or cytoplasmic location (Figure 5c and Figure S7).

The UCSC genome browser was used to analyze *Htt* exon1‐intron1 transcripts associated with TDP‐43 in the mouse striatum injected with AAV‐WT‐TDP‐43 or AAV‐cytoplasmic TDP‐43. The results showed that both WT‐TDP‐43 and cytoplasmic TDP‐43 were associated with the mRNA‐intron cluster, including the *Htt* exon1‐intron1 transcripts, which were enriched in (TG)_RIP_ region (Figure 5d and Figure S8). Furthermore, the fluorescent in situ hybridization (FISH) staining demonstrated the direct binding of pathologic TDP‐43 to the mouse *Htt* intron1 in the cytoplasm of HD KI mouse (Figure 5e), but the normal TDP‐43 remained in the nucleus in WT mouse striatum (Figure S9). To better examine whether the increased exon1‐intron1 transcripts are bound to cytoplasmic TDP‐43 and localized in the cytoplasm, we used probe‐*Htt* (Table S1), which targets In1b in intron1 to perform fluorescent FISH of the endogenous *Htt* exon1‐intron1 mRNAs in Neuro‐2a cells that were transfected with WT‐TDP‐43 or cytoplasmic TDP‐43 (Figure 5f and Figure S10). Although exon1‐intron1 transcripts are predominantly localized in the nucleus in WT‐TDP‐43 transfected cells, these transcripts are enriched in the cytoplasm of ΔNLS‐TDP‐43‐transfected cells (Figure S10), indicating that cytoplasmic TDP‐43 binds these transcripts and brings them to the cytoplasm.

### 2.4 Cytoplasmic TDP‐43 brings *Htt* exon1‐intron1 transcripts to the ribosome

**FIGURE 6:**
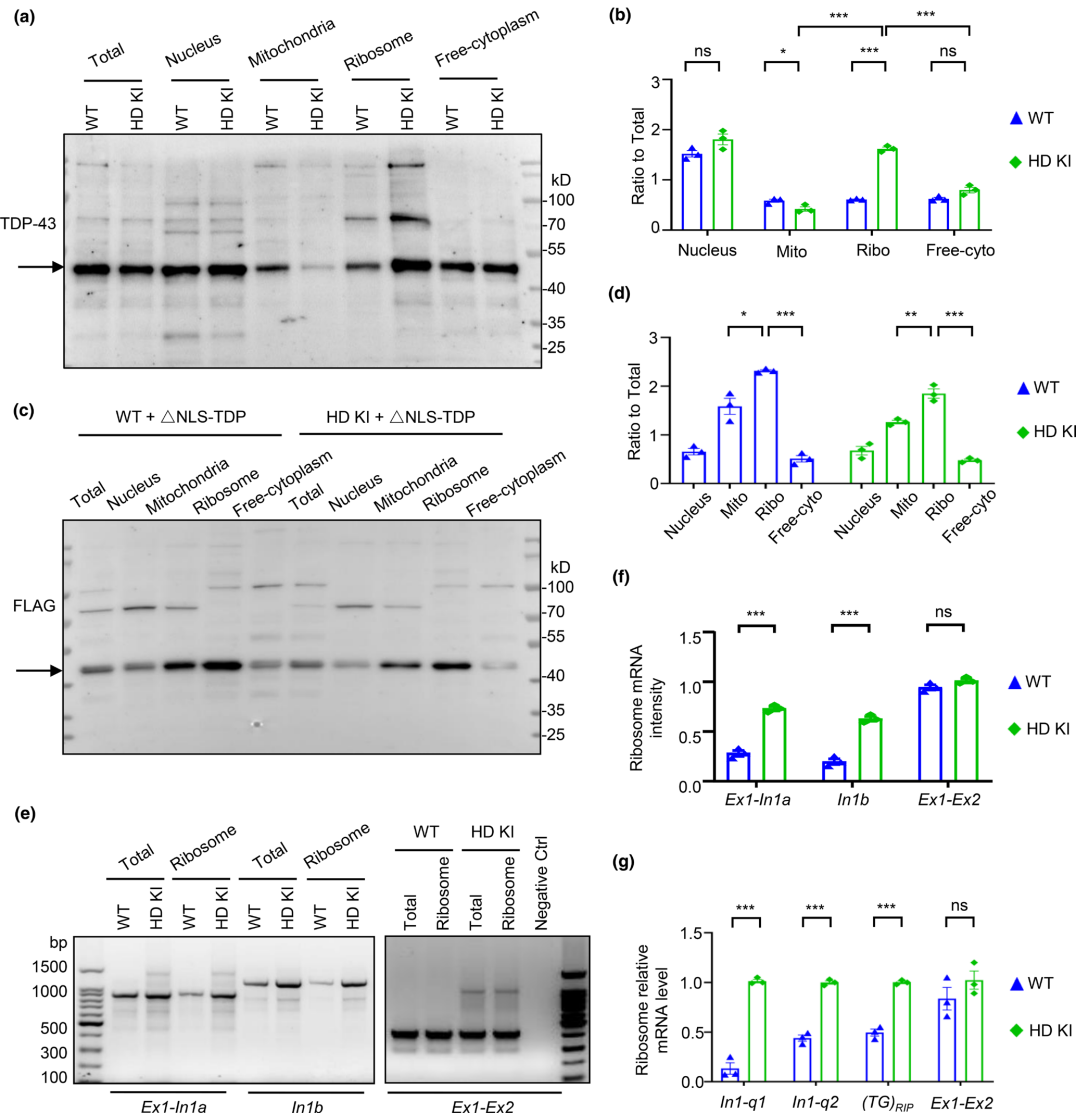
Cytoplasmic TDP‐43 promotes the binding of un‐spliced *Htt* exon1 intron1 transcripts to the ribosomes. (a) Western blotting analysis showed an increase of TDP‐43 (arrow) on the ribosome, in the HD KI mouse striatum as compared with WT mouse. (b) The relative levels of the distributed endogenous TDP‐43 on the ribosome. The data are mean ± SEM (*n* = 3 animals per group, 6 months old; ***p < 0.001; *p < 0.05; ns, non‐significant). (c) Western blotting analysis shows that the expression of cytoplasmic TDP‐43 (ΔNLS‐TDP‐43), which was detected by anti‐FLAG, is also distributed in the ribosome in both WT and HD KI mouse striatum. (d) The relative levels of the distributed exogenous cytoplasmic TDP‐43 in the ribosomes. The data are mean ± SEM (*n* = 3 animals per group, 3.5 months old; ***p < 0.001; **p < 0.01; *p < 0.05). (e) The semi‐PCR detection of the amplified Ex1‐In1a, In1b regions of *Htt* intron1 in the total homogenates or purified ribosome fraction showing that the HD KI striatum ribosomes in 6 months old, contain more *Htt* intron transcripts than WT mice. (f) The analysis of the different pre‐mRNA intensity in (e) of the amplified Ex1‐In1a, In1b and the maturity mRNA consisting of exon1 and exon2 regions of *Htt* in the purified ribosome fraction of WT and HD KI mice striatum. Note that in the ribosome fraction of HD KI striatum, more *Htt* intron transcripts were found than WT mouse, but without influence on maturity *Htt* mRNA level. The data are mean ± SEM, and obtained from three independent experiments (*n* = 3 animals per group, 6 months old; ***p < 0.001; ns, not significant). (g) The qPCR analysis of the different Htt intron transcript regions including In1‐q1, In1‐q2 and (TG)_RIP_ and mature mRNA constituted of exon1 and exon2, in the purified ribosome fraction. Note that the HD KI mouse ribosomes contain more un‐spliced intron1 transcripts than WT mouse ribosomes without influence on mature *Htt* mRNA level. The data are mean ± SEM, and obtained from three independent experiments (*n* = 3 animals per group, 6 months old; ***p < 0.001; ns, not significant).

Because exon1 Htt is increased in the mouse brain expressing cytoplasmic TDP‐43, we next explored whether cytoplasmic TDP‐43 promotes the binding of *Htt* exon1‐intron1 transcripts to the ribosome for translation after they are brought to the cytoplasm by cytoplasmic TDP‐43. We performed the subcellular fractionation experiment to compare the distribution of TDP‐43, and confirmed that TDP‐43 was distributed in the ribosome and that this ribosomal distribution was increased in HD KI mouse striatum (Figure 6a,b) and was also elevated by cytoplasmic TDP‐43 in either WT or HD KI mouse striatum (Figure 6c,d and Figure S11). Consistently, RT‐PCR revealed that more *Htt* exon1‐intron1 transcripts (Ex1‐In1a and In1b) were in the purified ribosomes fraction in the HD KI mouse striatum as compared with that in WT mice, whereas the normally spliced *Htt* transcripts (Ex1‐Ex2) without amplifying the CAG repeats showed similar in WT and KI mouse brains (Figure 6e,f).

**FIGURE 7:**
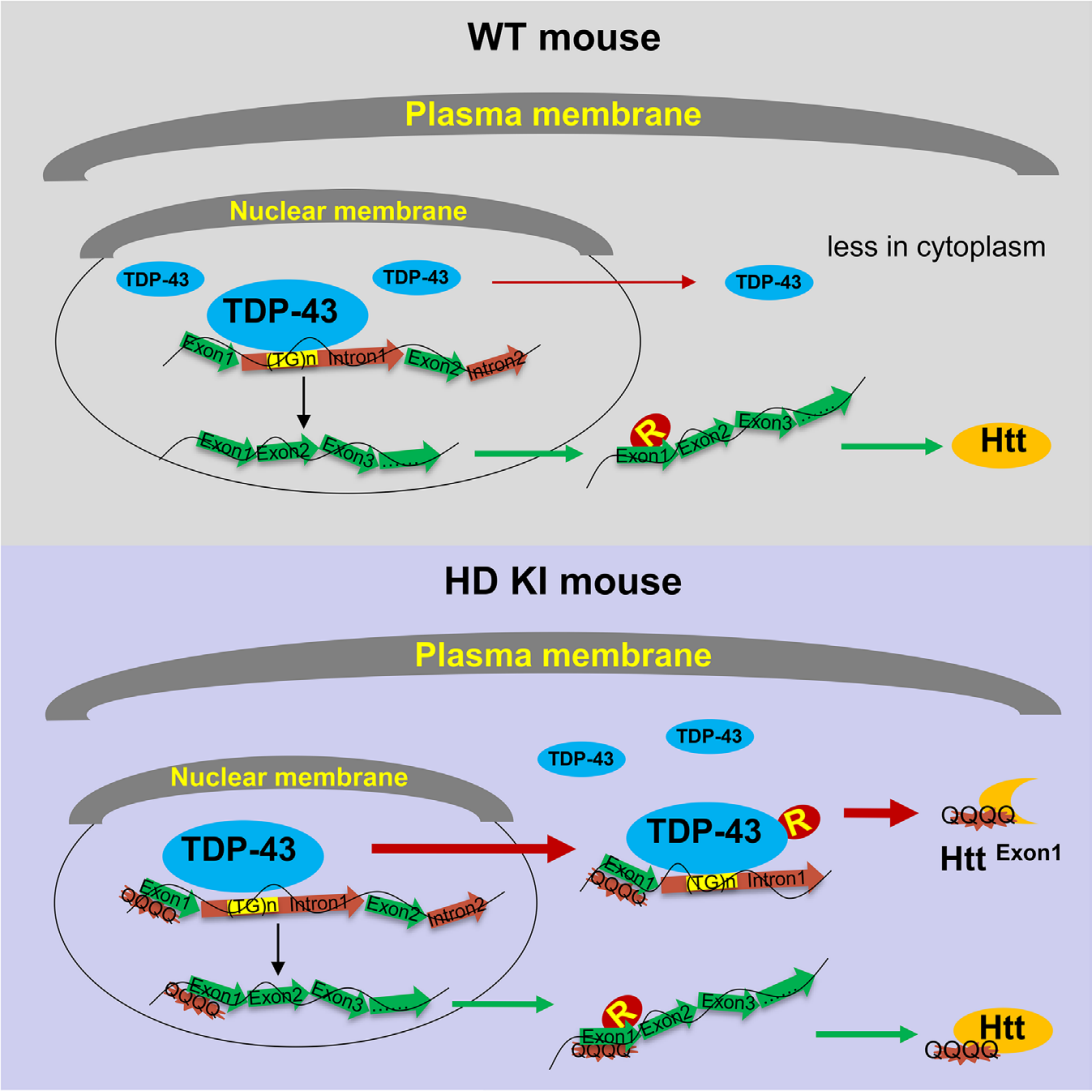
A proposed model for the aberrant generation of Htt‐exon1 mediated by the transport of cytoplasmic TDP‐43 in HD KI mice. In normal conditions, the majority of TDP‐43 is primarily localized in the nucleus and can bind to the GU/UG motif present in the intron1 of *Htt* pre‐mRNA, thereby regulating the processing of pre‐mRNA. However, in the HD KI mouse brain, the accumulation of cytoplasmic TDP‐43 leads to the translocation of mutant *Htt* pre‐mRNA, which is bound by TDP‐43, from the nucleus to the cytoplasm. Consequently, translation of the pre‐mRNA occurs in the cytoplasm, resulting in the production of toxic Htt exon1 protein.

We also performed the quantitative PCR analysis of these mouse striatum to evaluate the level of *Htt* exon1‐intron1 using PCR primers that could selectively amplify different regions (In1‐q1, In1‐q2 and (TG)_RIP_) of exon1‐intron1 and normally spliced *Htt* mRNA (Ex1‐Ex2). These exon1‐intron transcripts (In1‐q1, In1‐q2 and (TG)_RIP_) were more abundant in ribosomes from HD KI mouse striatum, whereas the matured *Htt* transcripts (Ex1‐Ex2) showed no significant change between the WT and KI mouse brains (Figure 6g). Because the HD KI mouse brain displays more abundant cytoplasmic TDP‐43, the above findings also support the idea that cytoplasmic TDP‐43 binds aberrant *Htt* exon1‐intron1 transcripts and brings them to the cytoplasmic ribosome for translation, resulting in the increased level of mutant exon1 Htt (Figure 7).

## 3 DISCUSSION

Cytoplasmic TDP‐43 accumulation is a hallmark of ALS/FTLD and has been observed in several other neurodegenerative diseases, such as Alzheimer's disease, Parkinson's disease and Huntington's disease (Chang et al., 2016; Ling et al., 2013; Ling, Hardy, & Zetterberg, 2015; Markopoulou et al., 2008), yet its contribution to these neurodegeneration remains poorly understood. TDP‐43 is involved in various RNA processing steps from splicing to translational regulation, providing multiple levels of regulation of gene expression such that its dysfunction results in gene expression dysregulation in diseases (Gao et al., 2018; Prasad et al., 2019). TDP‐43 binds to various RNA substrates and possesses two RNA‐recognition motifs (RRMs) that are involved in RNA binding (Buratti et al., 2001; Lukavsky et al., 2013). For the binding of TDP‐43 to various types of RNAs, it seems that multiple RRMs are required to confer specificity to highly structured RNA, and additional modules may collaborate for target binding (Auweter et al., 2006; Balcerak et al., 2019; Jankowsky & Harris, 2015; Lunde et al., 2007). The spatial arrangement of multiple RRMs in DNA/RNA binding proteins exerts steric effects on the substrate‐binding site, thereby controlling the specificity of the substrate's nucleotide sequences (Furukawa et al., 2016; Loughlin & Wilce, 2019). This phenomenon may be particularly relevant when binding to endogenous RNA. Furthermore, TDP‐43 associates with several cytoplasmic complexes including translational machinery (Coyne et al., 2015; Freibaum et al., 2010; Russo et al., 2017; Sephton et al., 2011) and stress granules (Colombrita et al., 2009; Dewey et al., 2011; Liu‐Yesucevitz et al., 2010; McDonald et al., 2011), suggesting a role for TDP‐43 in translation, as predicted by the ribostasis hypothesis (Ramaswami et al., 2013). The mislocalization of cytoplasmic TDP‐43 is likely to be detrimental to cellular function. For example, puromycin incorporation experiments in SH‐SY5Y neuroblastoma cells showed that increased cytoplasmic TDP‐43 reduces global translation through interactions with RACK1 on polyribosomes (Russo et al., 2017). On the other hand, polysome fractionation experiments in a human cell model showed that ALS associated mutant TDP‐43^A315T^ acts as a positive regulator of translation for a subset of specific mRNAs of Camta1, Mig12, and Dennd4A genes (Neelagandan et al., 2019). In diseases, TDP‐43 is also depleted from the nucleus, causing splicing defects, depression of cryptic exons (Brown et al., 2022; Ling, Pletnikova, et al., 2015; Ma et al., 2022), and increased retrotransposon expression (Krug et al., 2017; Liu et al., 2019; Morera et al., 2019; Tam et al., 2019). Taken together these previous findings highlight a complex role for TDP‐43 in translational regulation that can act both as a translational activator and inhibitor. Although the mislocalization of TDP‐43 from the nucleus to the cytoplasm was seen in HD patient cells and mouse models (Sanchez et al., 2021; Schwab et al., 2008; Tada et al., 2012), its contribution to HD pathogenesis remains unknown.

In the current study, we found that the cytoplasmic TDP‐43 was also increased in the HD KI mouse brain. The toxic effect of cytoplasmic TDP‐43 in HD is evidenced by the exacerbating effects of overexpressed cytoplasmic TDP‐43 on the behavioral phenotypes of HD KI mice. Moreover, we discovered that expression of cytoplasmic TDP‐43 can increase the level of N‐terminal mutant Htt at 55 kDa, similar to exon1 mutant Htt that has been found in various HD mice. By immunoprecipitation of this N‐terminal Htt, we confirmed that it carries exon1 Htt amino acid sequences. These findings indicate that cytoplasmic TDP‐43 can increase the expression of mutant exon1 Htt, a key pathological Htt fragment that is present in various HD mouse models (Yang et al., 2020), to contribute to HD pathogenesis.

Our findings also provide new clues to how cytoplasmic TDP‐43 can increase the toxic exon1 mutant Htt. We found that TDP‐43 can bind to the transcripts of exon1‐ intron1 of *Htt*, which have been found to be produced by aberrant splicing mechanisms (Arnold et al., 2013; Deshaies et al., 2018; Jiang et al., 2017). This binding could stabilize the aberrant *Htt* transcripts, then the transcripts are translated in the cytoplasm. Thus, when TDP‐43 is translocated to the cytoplasm, it can bring the aberrant *Htt* transcripts to the cytoplasm to promote their translation and generation of exon1 Htt protein product. In support of this idea, both TDP‐43 and *Htt* transcripts are found to be enriched in the ribosome, an essential step for the initiation of protein translation. Several groups have reported that the HD human brains also exhibit the cytoplasmic TDP‐43 (Sanchez et al., 2021; Schwab et al., 2008; Tada et al., 2012). The coding region of the human *HTT* gene shares over 80% nucleotide sequence identity with the mouse *Htt* gene. As there is a 56‐base pair GU/UG repeat in intron 1 of the human *HTT* gene (between base pairs 8856 and 8911), it remains to be investigated whether TDP‐43 binds to the mutant *HTT* intron region in human brains and promotes the cytoplasmic expression of mutant HTT exon1.

Another interesting issue that remains to be investigated is why nuclear TDP‐43 is moved to the cytoplasm under various pathological conditions. The cytoplasmic localization of TDP‐43 appears to be species‐dependent, as the majority of mouse models expressing mutant TDP‐43 show the predominant nuclear distribution of TDP‐43 (Mitchell et al., 2015; Philips & Rothstein, 2015; Shan et al., 2010; Wegorzewska et al., 2009; Weishaupt et al., 2016; Wils et al., 2010), in contrast to the cytoplasmic accumulation of TDP‐43 in the patient brains (Cairns et al., 2007; Lagier‐Tourenne & Cleveland, 2009; Neumann et al., 2006). Our previous studies suggest that the primate‐specific caspase‐4 cleaves TDP‐43 and removes the nuclear‐localization signals to generate cytoplasmic TDP‐43 (Yin et al., 2019). Consistently, caspase‐4 is upregulated under cellular stress and many pathological conditions (Hitomi et al., 2004; Martinez et al., 2017; Oakes & Papa, 2015). In HD mice, mutant Htt‐mediated neuropathology may initiate other cellular stress that can promote the cytoplasmic distribution of TDP‐43 directly. The consequent effects of cytoplasmic TDP‐43 appear to be broad to affect many cellular functions (Chhangani et al., 2021; Lee et al., 2021; Prasad et al., 2019). The findings in the current studies highlight a new pathogenic role of cytoplasmic TDP‐43 in transporting un‐spliced mRNA to the cytoplasmic ribosomes for the generation of an abnormal protein product.

In conclusion, under the normal conditions, TDP‐43 mainly or mostly exists in the nucleus and could bind to the GU/UG motif of *Htt* intron1 pre‐mRNA and regulate the pre‐mRNA mature processing. However, when TDP‐43 is accumulated in the HD KI mouse brain, the *Htt* pre‐mRNA that binds TDP‐43 will be moved from the nucleus to the cytoplasm. In the case of HD, mutant exon 1 *Htt* with an expanded CAG repeat will be translated in the cytoplasm, resulting in the generation of toxic Htt exon1 protein. Given that TDP‐43 binds various types of nuclear transcripts, the translocation of nuclear TDP‐43 to the cytoplasm could influence cellular function via the above suggested mechanism.

## 4 MATERIALS AND METHODS

### 4.1 Ethics statement

All subjects involved in the study signed informed written consent. Procedures were approved by the ethics committee of Jinan University (Approval No. IACUC‐20210220‐06).

### 4.2 Animal husbandry

All animal procedures were approved by the Institutional Animal Care and Use Committee of Jinan University. Full‐length mutant *Htt* knock‐in (140Q) (HD KI) mouse expressing human Exon1 *HTT* consisting of 140 CAGs in the endogenous mouse *Htt* gene were housed in the Division of Animal Resources at Jinan University on a 12‐h light /dark cycle. Heterozygous 140Q HD KI mice were then produced by mating male chimeric mice with female wild‐type C57BL/6J mice. All procedures and husbandry followed the NIH Guide for the Care and Use of Laboratory Animals. Genomic DNA was isolated from mouse tails, and PCR genotyping was used for screening HD KI mice. The forward and reverse primers (hHD25‐F and hHD177‐R, see Table S1) flanking the CAG repeat were used for PCR to amplify human Exon1 *HTT*.

### 4.3 Cells line

Mouse neuroblastoma (Neuro‐2a) cells were cultured in a complete culture medium consisting of Minimum Essential Medium (MEM, 41090036, Gibco) supplemented with 10% fetal bovine serum (FBS) and 1% penicillin/ streptomycin solution. Cells (2 × 10^5^ / well) were placed in the 12‐well culture plate pre‐treated with polylysine solution. For expression of exogenous WT‐ or ΔNLS‐TDP‐43, the cells were transfected with pRK‐WT‐ or pRK‐ΔNLS‐TDP‐43 plasmid, and pRK backbone as the vehicle control, using Lipofectamine 3000 Reagent (L3000001, Invitrogen) according to the manufactural protocol. After 2–4 days, transfected cells were subjected to immunofluorescent staining, fluorescence probe in situ hybridization, and western blotting.

### 4.4 Total RNA extraction and Q‐PCR

Frozen or fresh mice brains were used for purifying mRNA with trizol (1 mL for 50–100 mg brain tissue) before homogenization. One μg of total RNA was used for reverse transcription reactions using the PrimeScript TM RT reagent Kit (Takara, #RR047A). The synthesized cDNA was used for Q‐PCR with 2x SYBR Green PCR Master Mix (QuantiNova SYBR Green PCR Kit, #208052). For analysis, the 2^ΔΔCt^ method was used to calculate the relative fold gene expression of samples. The pre‐mRNA introns of the *Htt* gene were amplified by semi‐RT‐PCR primers Ex1‐In1a, In1b and Ex1 + Ex2, or real‐time qPCR primers In1‐q1, In1‐q2 and Ex1‐Ex2. The housekeeping gene β‐actin served as a control for quantitative real‐time PCR. The relative values of PCR results were normalized by β‐actin levels before calculations by GraphPad Prism8. All the primers are listed in Table S1.

### 4.5 Surgical procedures

Briefly, mice were anesthetized using isoflurane delivered with a vaporizer and stabilized in a stereotaxic instrument (David Kopf Instruments). All surgical procedures were performed in a designated procedure room and in accordance with the Guidelines for the Care and Use of Laboratory Animals and biosafety procedures at Jinan University. AAV9‐UBC vector for expressing ΔNLS‐TDP‐43 or WT‐TDP‐43 under the UBC promoter was purchased from Guangzhou Packgene Biotechnology Ltd. The titers of viruses were confirmed by determining the number of packaged vector genomes using quantitative PCR. Then the viruses were injected into both sides of the mouse striatum according to the following coordinates: 0.55 mm rostral to bregma, 2.0 mm lateral to the midline, and 3.5 mm ventral from the dural surface. The injection was conducted using a Hamilton syringe and a syringe infusion pump (World Precision Instruments, Inc, Sarasota, Florida, USA) at a speed of 100 nL/min for 20 min. During the surgery, mice were monitored with breath rates, movement, and body temperature continuously. One month after injection, the behavioral tests were conducted, and the injected mice were sacrificed by deep anesthesia with inhalation of isoflurane, and their brain tissues were isolated for western blotting.

### 4.6 Foot‐printing analysis

The CatWalkTM XT gait system (CatWalk for abbreviation), which consists of an enclosed walkway on a glass plate allowing a rodent to move from one side to the other freely, was applied for mice foot‐printing analysis. The CatWalk system includes a high‐speed digital camera beneath the walkway with a sample rate of 100 frames/s. When the foot of a rodent touches the glass plate of the walkway, the area touched will scatter the glass plate's internal green light and the scattered green light can be caught by the camera. Before foot‐printing analysis, animals were trained to make uninterrupted runs within 5 s. Measurements were executed after AAV‐ΔNLS‐TDP‐43 injection. Each mouse was placed on the walkway repeatedly at intervals of at least 10 min to complete three independent uninterrupted runs.

### 4.7 Western blotting

For western blotting analysis, cultured cells and mice brain tissues were lysed in ice‐cold RIPA buffer (50 mM Tris, pH 8.0, 150 mM NaCl, 1 mM ethylenediaminetetraacetic acid pH 8.0, 1 mM ethylene glycol tetraacetic acid, pH 8.0, 0.1% sodium dodecyl sulfate, 0.5% deoxycholate and 1% Triton X‐100) containing Halt protease inhibitor cocktail (Thermo Scientific) and PMSF. The lysates were sonicated and incubated on ice for 30 min and then centrifuged at 12000× g for 3 min at 4°C. Equal amounts of proteins from the whole lysates determined by BCA assay were subjected to western blotting analysis with appropriate primary antibodies (Htt‐EM48, MAB5374, Sigma‐Aldrich, 1:100; Htt‐4C8, MAB2166, Sigma‐Aldrich, 1:2000; Htt‐1C2, MAB1574, Sigma‐Aldrich, 1:1000; Vinculin, MAB3574, Sigma‐Aldrich, 1:3000; FLAG, F1804, Sigma‐Aldrich, 1:2000; TDP‐43, 12892‐1‐AP, Proteintech, 1:2000; p21^Cip1/Waf1^, ab227443, Abcam, 1:1000; Sirt1, HY‐P80319, MedChemExpress, 1:2000; LC3B, 2775S, Cell Signaling Technology, 1:2000; Beclin1, 3738, Cell Signaling Technology, 1:2000). All secondary antibodies were purchased from Jackson Immuno‐Research Laboratories. To quantify the ratios of target bands to the loading control, the blots were cut into strips to probe with their antibodies, thus the ratios could be obtained from the same blot.

### 4.8 Immunoprecipitation

Brain tissues (50 mg) from WT and HD KI mice were homogenized in 100 volumes (10 mg/mL) of cold 1% NP‐40 buffer (50 mM Tris, pH 7.4, 50 mM NaCl, 0.1% Triton X‐100, 1% NP‐40 with 1× protease inhibitors). Protease inhibitors at 1/100 (Sigma, P8340), PMSF, and phosphates inhibitors (2 mM sodium orthovanadate Na_3_VO_4_, Sigma, S6508) and 10 mM Sodium Fluoride (NaF, Sigma, S7920) were added to the homogenization buffer right before use. The brain tissue was cut into small pieces before putting it into the homogenization tube and stroked with the pistol 20 times on the ice. The samples (in an ice bath) were sonicated for 20 s after homogenization to break the genomic DNA. The homogenates were then incubated at 4°C for 30 mins with rocking at 4°C and centrifugated at 12000 × g for 10 mins. The supernatant was precleared with protein A agarose beads (Sigma, P1406), then the samples were immunoprecipitated with anti‐Htt (EM48), TDP‐43 antibody, or IgG control at 4°C overnight. Protein A agarose beads were added to capture the target proteins for 2 h at 4°C. Ice‐cold lysis buffer was used to wash beads three times. Proteins from the compound and input were subjected to Western blotting or mass spectrometry.

### 4.9 Mass spectrometry

The brain tissues of WT and HD KI mice were homogenized and immunoprecipitated using the EM48 antibody that can especially recognize the exon1 of Htt. The immunoprecipitated products were separated by SDS‐PAGE, and the gel was stained by coomassie blue. The target peptides or proteins, located between 55 and 70 kDa in coomassie blue staining gel, were collected and sent to Shenzhen BGI for protein mass spectrometry analysis.

### 4.10 Nuclear and cytoplasmic protein extraction

The brain tissues of WT and HD KI mice were harvested and separated nuclear and cytoplasmic proteins. Nuclear and cytoplasmic protein extraction was conducted using the Nuclear and Cytoplasmic protein extraction Kit (P0027, Beyotime), according to the manufacturer's instructions and then analyzed using western blotting.

### 4.11 Immunohistochemistry staining

Mice were anaesthetized with a peritoneal injection of Avertin (0.4–0.6 mg/g) and perfused with 0.9% NaCl followed by 4% paraformaldehyde (PFA). The brains were removed and post‐fixed in 4% PFA overnight at 4°C. The brains were transferred to 30% sucrose for 48 h to let the brain completely sink to the bottom of the 15 mL tube and then cut into 20 μm sections with the cryostat microtome (Thermo CRYOSTAR NX50) at −20°C. Mouse tissue sections were permeabilized with 0.3% Triton X‐100/PBS at room temperature for 1 h and then treated for antigen retrieval with citric acid buffer (10 mM citric acid, 0.05% Tween 20, pH 6.0) in 95°C water bath for 10 min. The mouse tissue sections were then blocked with 0.1% Triton X‐100/2% normal goat serum (NGS) and 3% BSA/1× PBS for 30 min. Brain sections at 20 μm were incubated with primary antibody (TDP‐43, 12,892‐1‐AP, Proteintech, 1:2000) at 4°C. HRP/DAB Detection IHC kit and DAB kit for immunodetection were used. Images were acquired using a Zeiss microscope (AXIO Imager.A2) with a digital camera (Axiocam 506 color ZEISS) and ZEN 2.3 software.

### 4.12 Immunofluorescence staining

After discarding the culture medium, the cells were washed twice with PBS for 3 min each time. The cells were added with 500 μL 0.4% PFA solution for 20 min, and washed with PBS 3 times, 3 min each time. The fixed cells were permeabilized with 0.3% Triton X‐100 at room temperature for 1 h and then blocked with 3% BSA for 30 min. The cell samples were incubated with primary antibodies (FLAG, F1804, Sigma‐Aldrich, 1:2000; RPS6, 2317S, Cell Signaling, 1:100; Htt‐EM48, MAB5374, Sigma‐Aldrich, 1:100; NeuN, ab177487, Abcam, 1:1000; GFAP, ab7260, Abcam, 1:1000; Iba1, 17198S, Cell Signaling Technology, 1:1000) at 4°C overnight, followed by incubation with Alexa 488‐ or/and Taxes red 615‐conjugated secondary antibodies and DAPI nuclear dye. Images were acquired with a Zeiss microscope (AXIO Imager.A2) with a digital camera (Axiocam 506 color ZEISS) and ZEN 2.3 software.

### 4.13 RNA immunoprecipitation (RIP)

1 × 10^7^ Neuro‐2a cells were harvested by trypsinization and resuspended in 2 mL PBS, 2 mL nuclear isolation buffer (1.28 M sucrose; 20 mM MgCl_2_; 4% Triton X‐100; 40 mM Tris–HCl pH 7.5), and 6 mL sterile water on ice for 20 min (with frequent mixing). Nuclei were pelleted by centrifugation at 2500× g for 15 min. The nuclear pellet was resuspended in 1 mL RIP buffer (150 mM KCl, 5 mM EDTA, 0.5 mM DTT, 0.5% NP40, 25 mM Tris pH 7.4, 9 μg/mL leupeptin, 9 μg/mL pepstatin, 10 μg/mL chymostatin, 3 μg/mL aprotinin, 1 mM PMSF, 100 U/mL SUPERASin; Ambion). Resuspended nuclei were mechanically sheared using a Dounce homogenizer with 15–20 strokes. Nuclear membrane and debris were pelleted by centrifugation at 13400× g for 10 min. Antibody to TDP‐43 (12892‐1‐AP, Proteintech, 1:100) was added to the supernatant and incubated for 2 h at 4°C with gentle rotation. 40 μL of protein G beads were added and incubated for 1 h at 4°C with gentle rotation. Beads were pelleted at 2000× g for 30 s, the supernatant was removed, and beads were resuspended in 500 μL RIP buffer and repeated for a total of three RIP buffer washes, followed by one wash in PBS. Beads were resuspended in 1 mL Trizol. Coprecipitated RNAs were isolated and sent to Beijing Novogene (China) Biotechnology Ltd for library construction and sequencing on Illumina HiSeq 2500 or used for PCR analysis. Also, RT‐PCR for TDP‐43‐associated mRNAs was performed. Protein isolated using the beads was detected by western blotting. The UCSC genome browser (https://genome.ucsc.edu) was used to analyze and visualize the general transcript reads of the associated RNAs.

### 4.14 RNA fluorescence in situ hybridization (RNA‐FISH)

Cy3‐labeled RNA probes targeting *Htt* intron1 pre‐mRNA were designed and synthesized by Guangzhou IGE Biotechnology Ltd. RNA‐FISH was conducted using the Fluorescent in situ Hybridization Kit (Shanghai GenePharma Co., Ltd), according to the manufacturer's instruction. Images were acquired with a Zeiss microscope (AXIO Imager.A2) with a digital camera (Axiocam 506 color ZEISS) and ZEN 2.3 software.

### 4.15 Subcellular fractionations of brain tissues

Mouse brain tissues were homogenized for 25 strokes with a dounce homogenizer ice‐cold buffer (0.32 M sucrose, 15 mM Tris–HCl, 60 mM KCl, 15 mM NaCl, 5 mM EDTA, 1 mM EGTA, 0.02% NaN3, 2 mM ATP, pH 8.0) containing protease inhibitor and 100 μM PMSF. Ten percent lysates were stored as the total lysate sample. Nuclei and cellular debris were pelleted at 800× g for 5 min. The supernatant was transferred to a new tube and centrifuged at 20000× g for 30 min at 4°C, to obtain the mitochondria‐enriched pellet. Then the supernatant was then centrifuged at 100000× g for 30 min at 4°C to separate the ribosome‐enriched pellet and the soluble free‐cytoplasmic fraction. Crude nuclear pellets were washed four times with ice‐cold homogenization buffer to remove cytoplasmic contaminants. For nuclear purification, the pellets were re‐suspended in 374 μL of buffer (15 mM HEPES, 1.5 mM MgCl_2_, 0.2 mM EDTA, 0.5 mM DTT, 26% glycerol, pH 7.9) with 26 μL of 4.6 M NaCl to generate the final concentration at 300 mM NaCl, homogenized with 20 full strokes in Tefon homogenizer on ice, and sonicated for 10 s. The homogenized samples were kept on ice for 20 min and then centrifuged at 24000× g for 20 min at 4°C, to obtain the purified nuclear.

### 4.16 Quantification and statistical analysis

For biochemical and histological studies, we used at least three mice per group. Three independent experiments were done for figure presentations, and the representative results were shown in figures. Statistical significance was assessed using the two‐tailed Student's t‐test for comparison if there were only two groups. When analyzing multiple groups, we used one‐way ANOVA or two‐way ANOVA followed by Tukey's multiple comparisons test to determine statistical significance. A p‐value <0.05 was considered significant. Data represent the mean ± SEM. Calculations were performed with GraphPad Prism8 software.

## AUTHOR CONTRIBUTIONS

Xiao‐Jiang Li, Peng Yin and Dazhang Bai designed research. Dazhang Bai, Fuyu Deng, Qingqing Jia, Kaili Ou, Xiang Wang, Junqi Hou and Longhong Zhu performed research and analyzed data. Kaili Ou and Mingwei Guo assisted on the animal maintenance. Su Yang, Guohui Jiang and Shihua Li provided technical guidance. Xiao‐Jiang Li, Peng Yin and Dazhang Bai wrote the paper.

## FUNDING INFORMATION

This work was supported by the National Natural Science Foundation of China 32,270,564 (P.Y.), 82,394,422 (X.J.L.), 82,071,421 (S.H.L), 82,271,902 (S.H.L); Department of Science and Technology of Guangdong Province 2021ZT09Y007 (X.J.L.), 2018B030337001 (X.J.L.); Basic and Applied Basic Research of Guangdong Province 2022A1515011205 (P.Y.), 2023A1515010811 (P.Y.); the Doctoral Research Foundation of North Sichuan Medical College CBY21‐QD17 (D.B.).

## CONFLICT OF INTEREST STATEMENT

The authors declare that they have no competing interests.

## PERMISSION STATEMENT

We permit the right to Wiley and *Aging Cell* to license and reproduce the above information. We required no permissions for any data or figures produced in this manuscript.

## Supporting information


Data S1.

## Data Availability

The mass spectrometry sequencing data of the precipitated Htt have been deposited at https://doi.org/10.6084/m9.figshare.24072360, and are publicly available as of the date of publication.
