# Supplementary material for: Pathogenic TDP‐43 accelerates the generation of toxic exon1 HTT in Huntington's disease knock‐in mice
Source: Aging Cell. 2024 Aug 26;23(12):e14325. doi: 10.1111/acel.14325 (PMC11634733; doi:10.1111/acel.14325)
Supplement: Supplementary file 1 — Data S1. [file ACEL-23-e14325-s001.pdf]

Figure S1

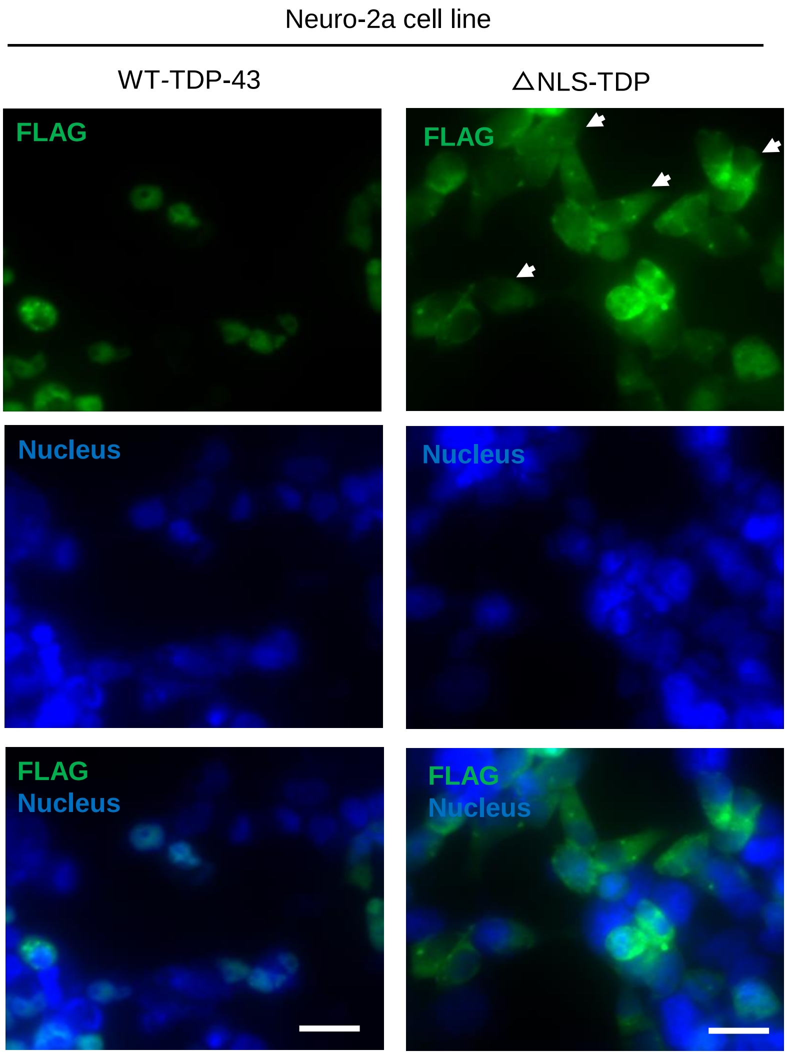

Neuro-2a cell line
WT-TDP-43
△NLS-TDP
FLAG
FLAG
Nucleus
Nucleus
FLAG
Nucleus
FLAG
Nucleus

Figure S2

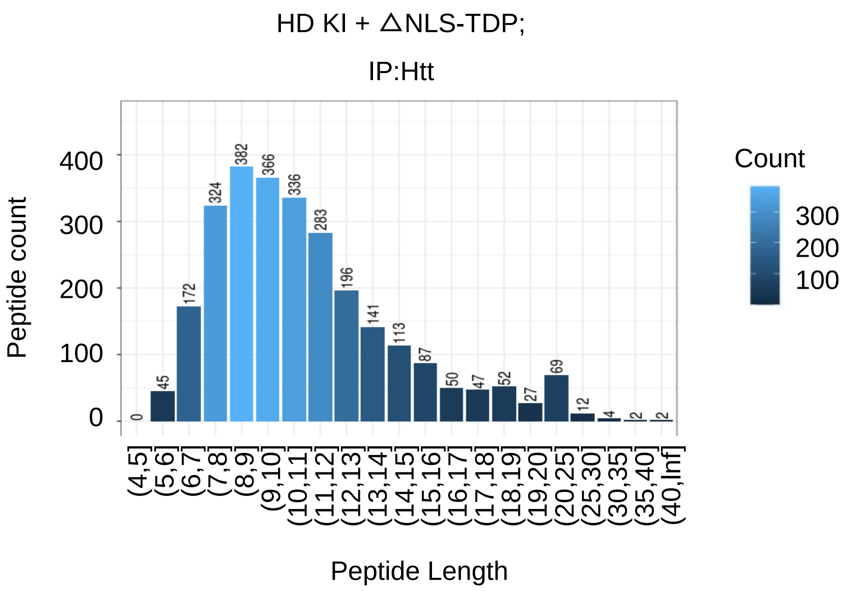

HD KI + △NLS-TDP;
IP:Htt
Peptide count
400
300
200
100
0
0
45
172
324
382
366
336
283
196
141
113
87
50
47
52
27
69
12
4
2
2
(4,5]
(5,6]
(6,7]
(7,8]
(8,9]
(9,10]
(10,11]
(11,12]
(12,13]
(13,14]
(14,15]
(15,16]
(16,17]
(17,18]
(18,19]
(19,20]
(20,25]
(25,30]
(30,35]
(35,40]
(40,Inf]
Peptide Length
Count
300
200
100

Figure S3

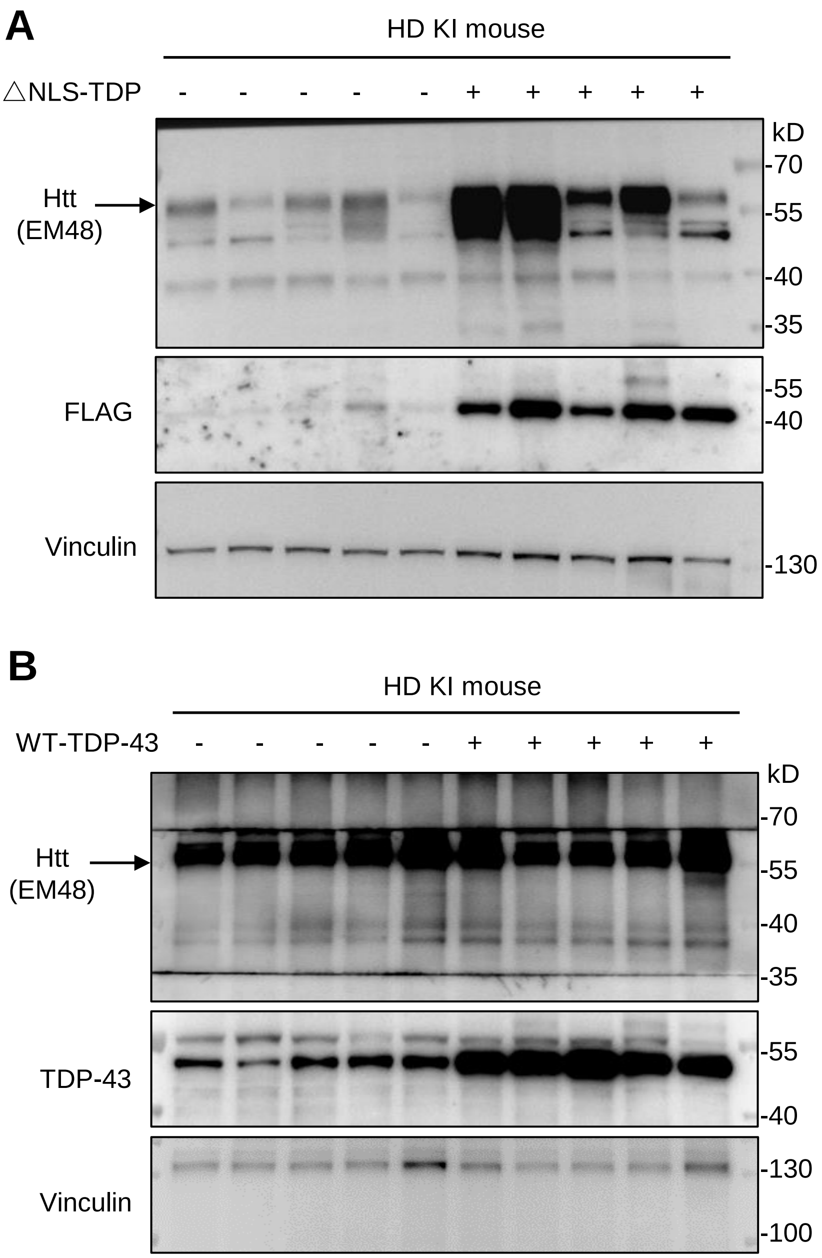
A
HD KI mouse
△NLS-TDP - - - - - + + + + +
kD
Htt (EM48)
FLAG
Vinculin
-70
-55
-40
-35
-55
-40
-130
B
HD KI mouse
WT-TDP-43 - - - - - + + + + +
kD
Htt (EM48)
TDP-43
Vinculin
-70
-55
-40
-35
-55
-40
-130
-100

Figure S4

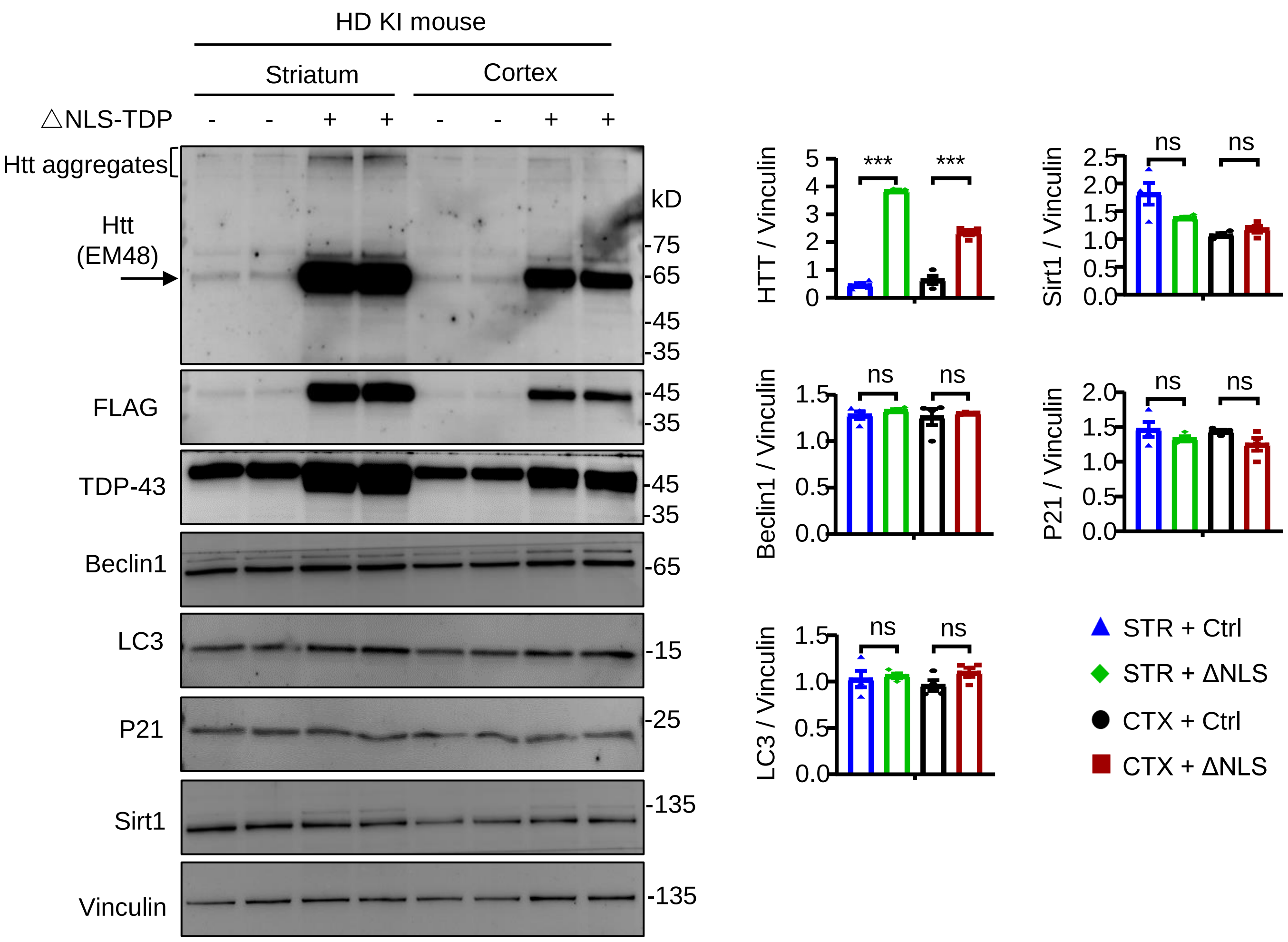
HD KI mouse
Striatum
Cortex
△NLS-TDP - - + + - - + +
Htt aggregates
Htt (EM48)
FLAG
TDP-43
Beclin1
LC3
P21
Sirt1
Vinculin
kD
-75
-65
-45
-35
-45
-35
-45
-35
-65
-15
-25
-135
-135
HTT / Vinculin
***
***
Sirt1 / Vinculin
ns
ns
Beclin1 / Vinculin
ns
ns
P21 / Vinculin
ns
ns
LC3 / Vinculin
ns
ns
STR + Ctrl
STR + ΔNLS
CTX + Ctrl
CTX + ΔNLS

Figure S5

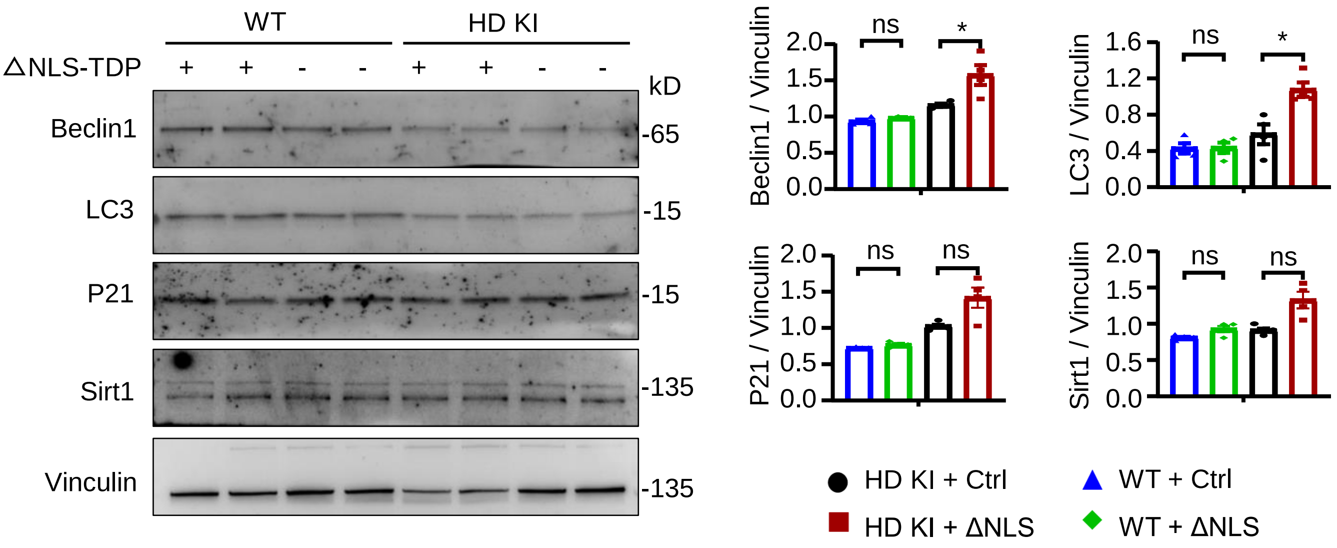

WT
HD KI
△NLS-TDP
+ + - - + + - -
kD
Beclin1
-65
LC3
-15
P21
-15
Sirt1
-135
Vinculin
-135
Beclin1 / Vinculin
ns
*
2.0
1.5
1.0
0.5
0.0
LC3 / Vinculin
ns
*
1.6
1.2
0.8
0.4
0.0
P21 / Vinculin
ns
ns
2.0
1.5
1.0
0.5
0.0
Sirt1 / Vinculin
ns
ns
2.0
1.5
1.0
0.5
0.0
HD KI + Ctrl
HD KI + ΔNLS
WT + Ctrl
WT + ΔNLS

Figure S6

A

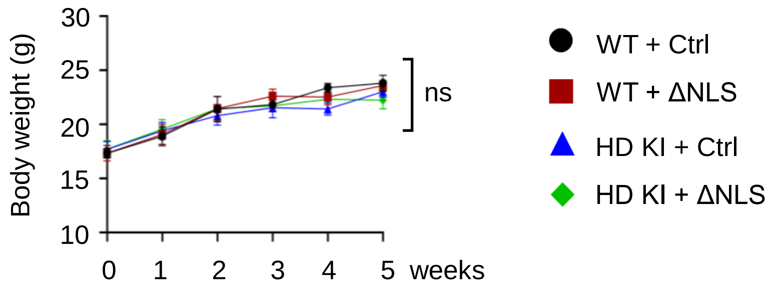

Body weight (g)
30
25
20
15
10
0
1
2
3
4
5
weeks
ns
WT + Ctrl
WT + ΔNLS
HD KI + Ctrl
HD KI + ΔNLS

B

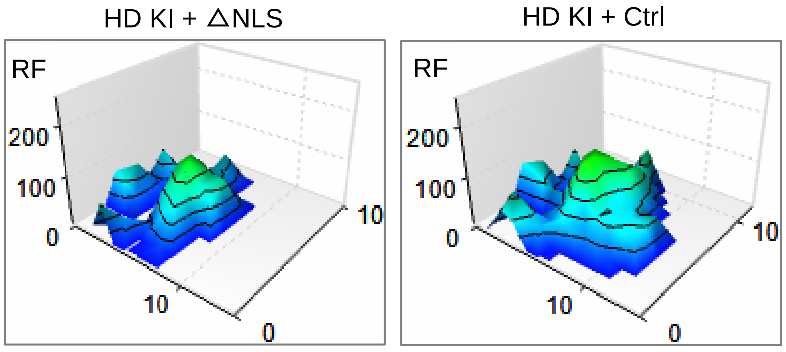

HD KI + ΔNLS
HD KI + Ctrl
RF
200
100
0
10
10
0

Figure S7

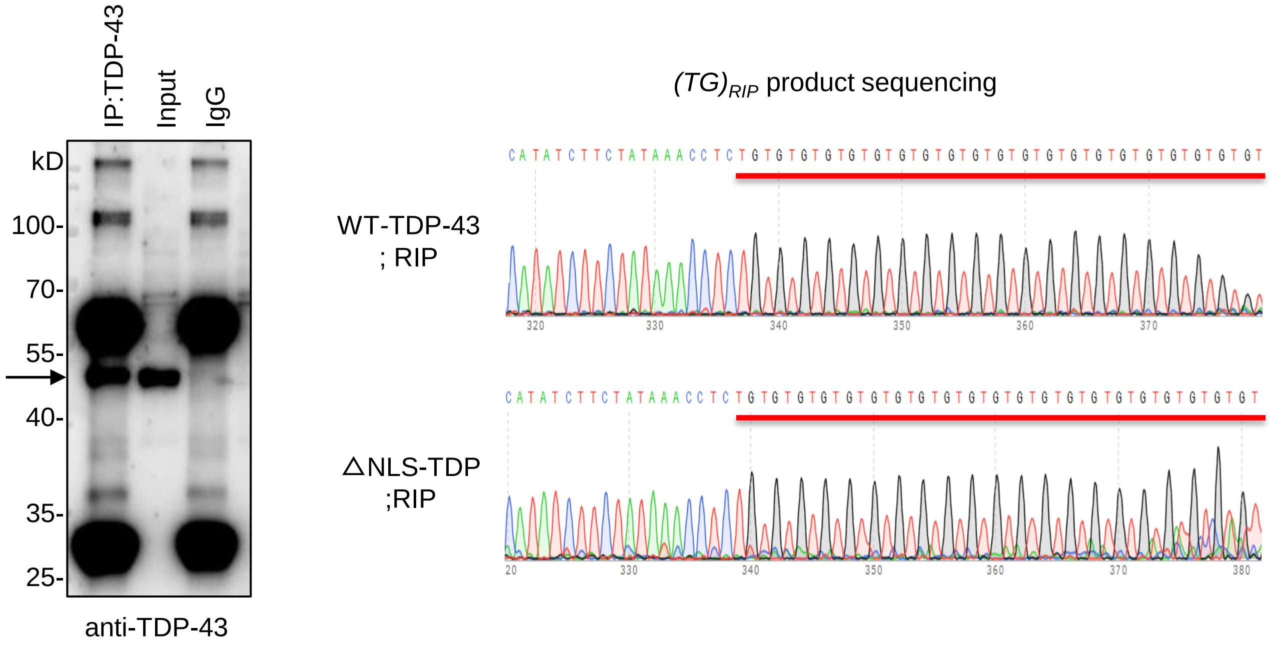

IP:TDP-43
Input
IgG
kD
100-
70-
55-
40-
35-
25-
anti-TDP-43
(TG)RIP product sequencing
WT-TDP-43
; RIP
△NLS-TDP
;RIP

Figure S8

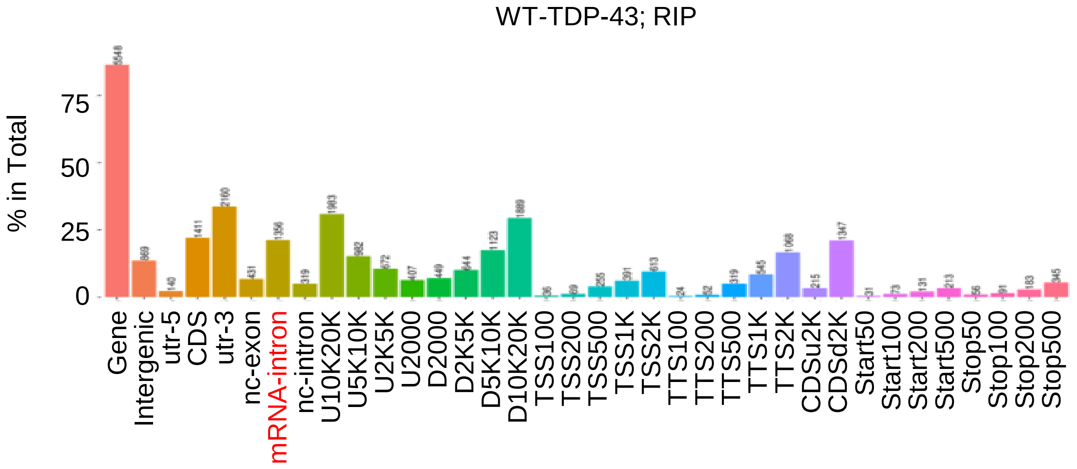
WT-TDP-43; RIP
% in Total
75
50
25
0
Gene
Intergenic
utr-5
CDS
utr-3
nc-exon
mRNA-intron
nc-intron
U10K20K
U5K10K
U2K5K
U2000
D2000
D2K5K
D5K10K
D10K20K
TSS100
TSS200
TSS500
TSS1K
TSS2K
TTS100
TTS200
TTS500
TTS1K
TTS2K
CDSu2K
CDSd2K
Start50
Start100
Start200
Start500
Stop50
Stop100
Stop200
Stop500

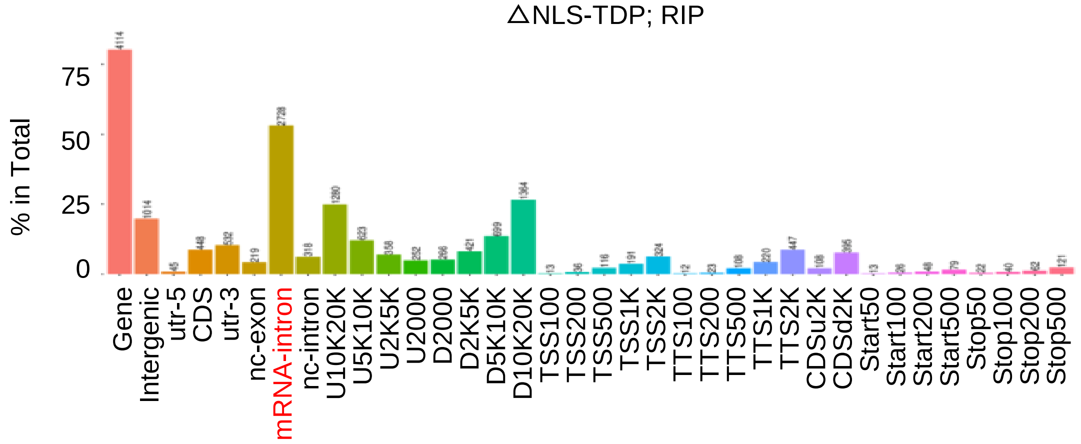
△NLS-TDP; RIP
% in Total
75
50
25
0
Gene
Intergenic
utr-5
CDS
utr-3
nc-exon
mRNA-intron
nc-intron
U10K20K
U5K10K
U2K5K
U2000
D2000
D2K5K
D5K10K
D10K20K
TSS100
TSS200
TSS500
TSS1K
TSS2K
TTS100
TTS200
TTS500
TTS1K
TTS2K
CDSu2K
CDSd2K
Start50
Start100
Start200
Start500
Stop50
Stop100
Stop200
Stop500

Figure S9

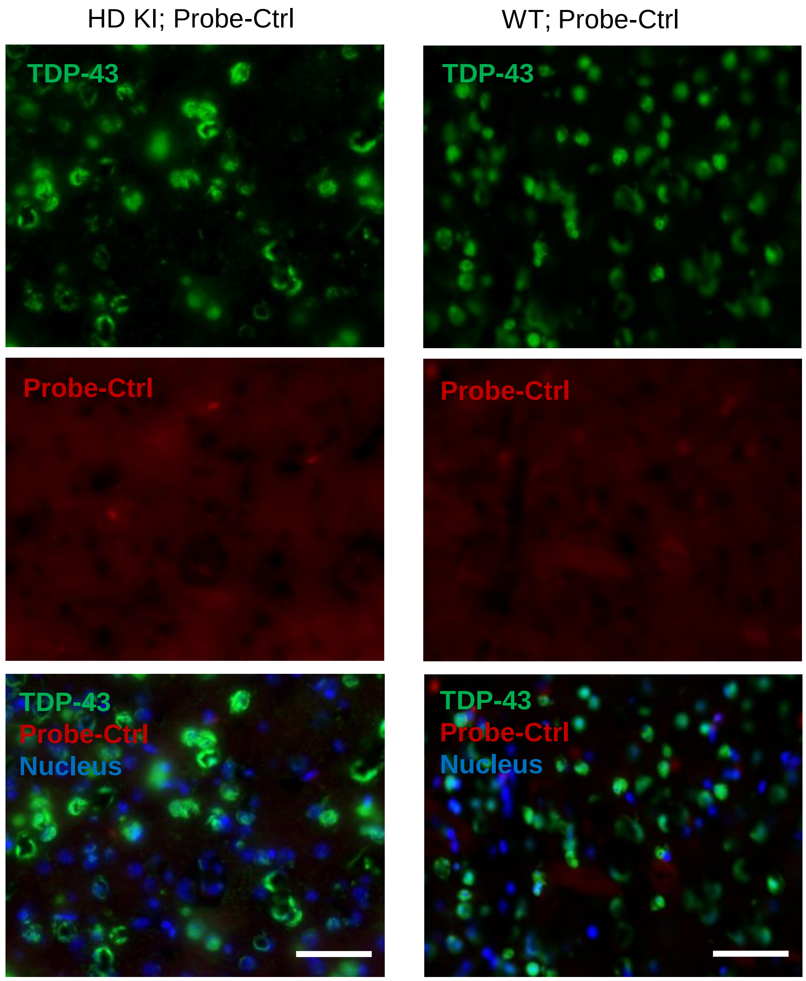
HD KI; Probe-Ctrl
WT; Probe-Ctrl
TDP-43
Probe-Ctrl
TDP-43
Probe-Ctrl
Nucleus

Figure S10

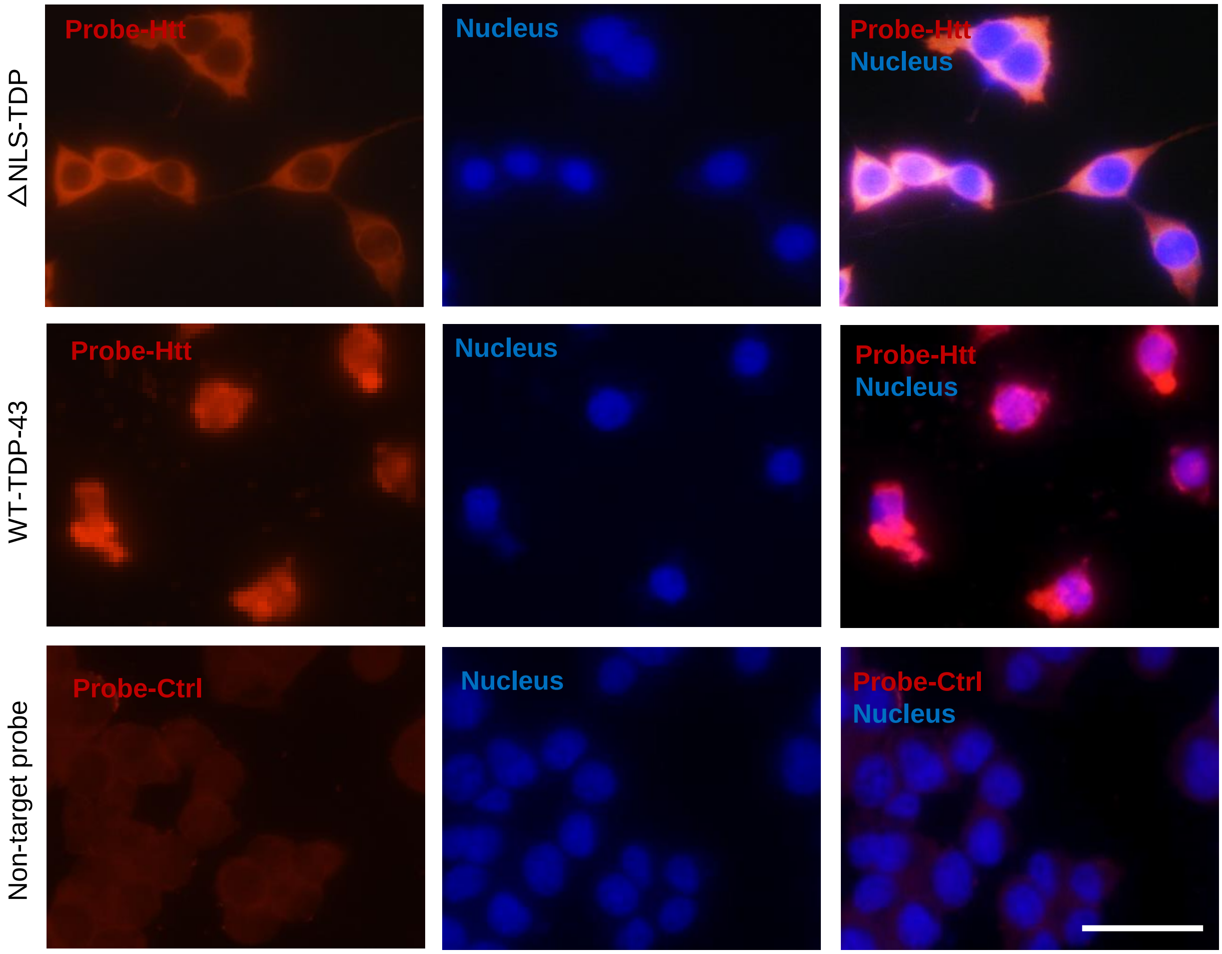

ΔNLS-TDP
WT-TDP-43
Non-target probe
Probe-Htt
Nucleus
Probe-Htt
Nucleus
Probe-Htt
Nucleus
Probe-Htt
Nucleus
Probe-Ctrl
Nucleus
Probe-Ctrl
Nucleus

Figure S11

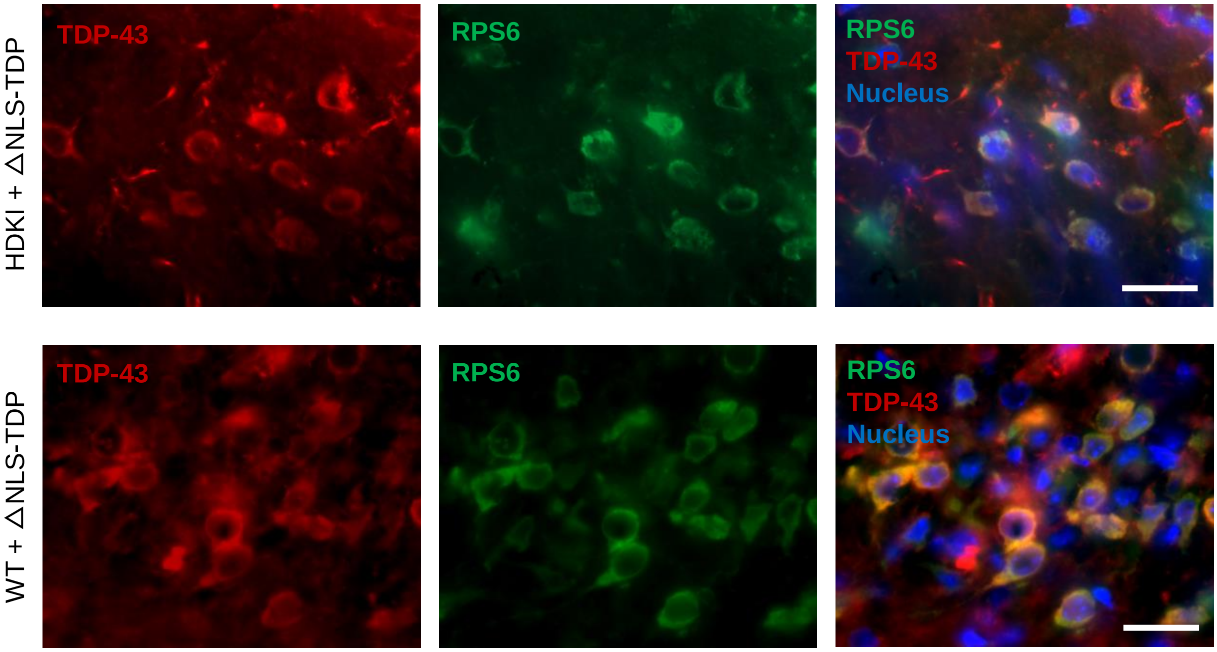
HDKI + △NLS-TDP
TDP-43
RPS6
RPS6
TDP-43
Nucleus
WT + △NLS-TDP
TDP-43
RPS6
RPS6
TDP-43
Nucleus

Table S1

Primers	Sequences
Probe-*Htt*	CUGUUUCAAUAUUCUCUCCAGUCACAAGCAG
hHD25-F	AAGGCCTTCGAGTCCCTCAAGT
hHD177-R	GAGGCAGCAGCGGCTGTGCCTG
Ex1-In1a-F	ATGGCAACCCTGGAAAAGCTGATGA
Ex1-In1a-R	GACAAATACAACTCATCCAACTGCC
In1b-F	AAGTACATGGTGAGTTACTTAGGTG
In1b-R	CTGGTGAATTTCAGTGTATCTTGTC
In1-q1-F	GTTTAGTTCTTTGCTGACATCTTAT
In1-q1-R	CACTCAGCATGGATTTCCAGGAAA
In1-q2-F	GGAGGAGAGTCCTAGAAATTCATTTC
In1-q2-R	CCCAGAGGCAGAGTCATGGTAATAA
$(TG)_{RIP}$-F	TGAGAGGAATTGTGGAAGTTTAC
$(TG)_{RIP}$-R	ACAGAACAATCTCTCTCTCTCTC
Ex1-Ex2-F	TCCGCCTCAACCCCCTCA
Ex1-Ex2-R	CTGAGAGACTGTGCCACAATG
